# In Vivo and In Vitro Metabolic Fate and Urinary Detectability of Five Deschloroketamine Derivatives Studied by Means of Hyphenated Mass Spectrometry

**DOI:** 10.3390/metabo14050270

**Published:** 2024-05-08

**Authors:** Fabian Frankenfeld, Lea Wagmann, Anush Abelian, Jason Wallach, Adeboye Adejare, Simon D. Brandt, Markus R. Meyer

**Affiliations:** 1 Department of Experimental and Clinical Toxicology, Institute of Experimental and Clinical Pharmacology and Toxicology, Center for Molecular Signaling (PZMS), Saarland University, 66421 Homburg, Germany; 2 Department of Pharmaceutical Sciences, Philadelphia College of Pharmacy, Saint Joseph’s University, Philadelphia, PA 19104, USA; 3 School of Pharmacy and Biomolecular Sciences, Liverpool John Moores University, Liverpool L3 3AF, UK

**Keywords:** new psychoactive substance, deschloroketamine, deschloro-*N*-ethyl-ketamine, deschloro-*N*-isopropyl-ketamine, deschloro-*N*-cyclopropyl-ketamine, deschloro-*N*-propyl-ketamine, metabolism, in vivo, in vitro, LC-HRMS/MS

## Abstract

Ketamine derivatives such as deschloroketamine and deschloro-*N*-ethyl-ketamine show dissociative and psychoactive properties and their abuse as new psychoactive substances (NPSs) has been reported. Though some information is available on the biotransformation of dissociative NPSs, data on deschloro-*N*-cyclopropyl-ketamine deschloro-*N*-isopropyl-ketamine and deschloro-*N*-propyl-ketamine concerning their biotransformation and, thus, urinary detectability are not available. The aims of the presented work were to study the in vivo phase I and II metabolism; in vitro phase I metabolism, using pooled human liver microsomes (pHLMs); and detectability, within a standard urine screening approach (SUSA), of five deschloroketamine derivatives. Metabolism studies were conducted by collecting urine samples from male Wistar rats over a period of 24 h after their administration at 2 mg/kg body weight. The samples were analyzed using liquid chromatography high-resolution tandem mass spectrometry (LC-HRMS/MS) and gas chromatography–mass spectrometry (GC-MS). The compounds were mainly metabolized by *N*-dealkylation, hydroxylation, multiple oxidations, and combinations of these metabolic reactions, as well as glucuronidation and *N*-acetylation. In total, 29 phase I and 10 phase II metabolites were detected. For the LC-HRMS/MS SUSA, compound-specific metabolites were identified, and suitable screening targets could be recommended and confirmed in pHLMs for all derivatives except for deschloro-*N*-cyclopropyl-ketamine. Using the GC-MS-based SUSA approach, only non-specific acetylated *N*-dealkylation metabolites could be detected.

## 1. Introduction

According to the European Monitoring Centre for Drugs and Drug Addiction (EMCDDA) in Europe, over 83 million adults have used illicit drugs at least once in their life. Data from the European Web Survey on Drugs reported that, in 2021, next to the use of lysergic acid diethylamide and new psychoactive substances (NPSs), 13% of those surveyed consumed ketamine [1]. Furthermore, the annual amounts of seized ketamine have been at relatively high levels since its peak in 2018, with over 1.7 tons seized [2]. Ketamine is known as an anesthetic and analgesic and is listed on the World Health Organization’s list of essential medicines [3,4]. Ketamine can also induce psychoactive and dissociative effects caused by the antagonism of *N*-methyl-D-aspartate receptors [5,6]. Structurally related compounds, such as phencyclidine (also known as PCP or angel dust), show similar effects that have been associated with their recreational use [6]. NPSs derived from ketamine are emerging on the drug market, with deschloroketamine (2-oxo-PCMe) first reported in the UK in 2015 [7] and deschloro-*N*-ethyl-ketamine (2-oxo-PCE) first reported in France in 2016 [8]. Both substances were patented by Stevens in 1961 [9] and are potent dissociative agents with ethanol-like effects in lower doses, with noticeable dissociative effects described for 2-oxo-PCMe and analgesic as well as dissociative effects described for 2-oxo-PCE [4]. Hájková et al. described the metabolism of 2-oxo-PCMe in rats after its subcutaneous administration [10], Larabi et al. studied the metabolism of 2-oxo-PCE in human liver microsomes (HLMs) [11], and Tang et al. reported cases of acute poisonings in humans associated with the intake of 2-oxo-PCE. These authors tentatively identified three metabolites of 2-oxo-PCE. However, detailed knowledge on the metabolism of 2-oxo-PCE has remained limited, supporting the importance of investigating the metabolism of emerging NPSs in the context of forensic and clinical toxicology [12].

**Figure 1:**
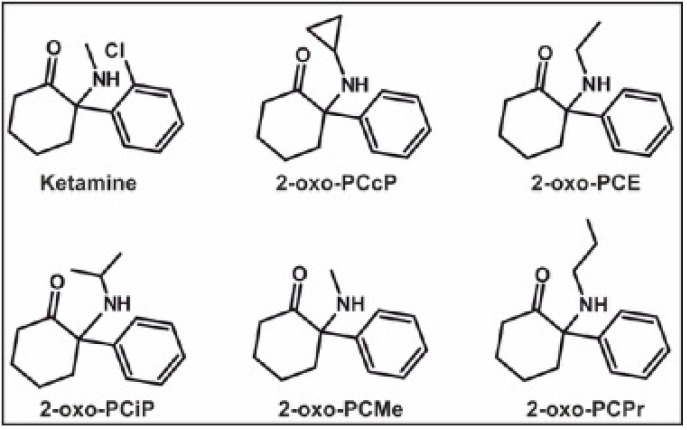
Structures of ketamine and deschloroketamine derivatives 2-oxo-PCcP, 2-oxo-PCE, 2-oxo-PCiP, 2-oxo-PCMe, and 2-oxo-PCPr.

Hence, this study aimed to investigate the in vivo metabolic fate of five deschloroketamine derivatives: deschloro-*N*-cyclopropyl-ketamine (2-oxo-PCcP), 2-oxo-PCE, deschloro-*N*-isopropyl-ketamine (2-oxo-PCiP), 2-oxo-PCMe, and deschloro-*N*-propyl-ketamine (2-oxo-PCPr, see Figure 1). Sample preparations were followed by an analysis using liquid chromatography high-resolution tandem mass spectrometry (LC-HRMS/MS) or gas chromatography coupled with mass spectrometry (GC-MS). Furthermore, in vivo phase I metabolites should be confirmed by human in vitro data after incubations using pooled HLMs (pHLMs). The limits of identification (LOI) in human urine samples, as well as standard urine screening approaches (SUSA) by LC-HRMS/MS and GC-MS, were investigated.

## 2. Materials and Methods

2-Oxo-PCcP hydrochloride, 2-oxo-PCE hydrochloride, 2-oxo-PCiP hydrochloride, 2-oxo-PCMe hydrochloride, and 2-oxo-PCPr were synthesized by the standard methods and analytically characterized by ^1^H and ^13^C nuclear magnetic resonance (NMR), high-performance liquid chromatography (HPLC) (>95% pure on HPLC and NMR), and high-resolution mass spectrometry (HRMS.) Acetonitrile (ACN), ammonium formate, dichloromethane (DCM), ethyl acetate, hydrochloric acid (HCl), isopropanol, methanol (MeOH), sodium hydroxide solution (NaOH), and all other chemicals were obtained from VWR International GmbH (Darmstadt, Germany). Diazepam-d5 was obtained from LGC Standards GmbH (Wesel, Germany). NADP^+^ was obtained from Biomol (Hamburg, Germany), superoxide dismutase, magnesium chloride (MgCl_2_), isocitrate, and isocitrate dehydrogenase were from Merck (Darmstadt, Germany). pHLMs (20 mg microsomal protein/mL, 330 pmol total CYP/mg protein) were obtained from Corning (Amsterdam, the Netherlands). All enzyme-containing preparations were thawed at 37 °C after delivery, aliquoted, snap frozen in liquid nitrogen and stored at −80 °C until use. Water was purified with a Millipore (Merck, Darmstadt, Germany) filtration unit, which purifies water to a resistance of 18.2 Ω × cm. Isolute HCX solid-phase extraction (SPE) cartridges (130 mg, 3 mL) were obtained from Biotage (Uppsala, Sweden).

### 2.1. Rat Urine Samples

Six male Wistar rats (Charles River, Sulzfeld, Germany) were used for our metabolism studies in accordance with German laws for animal protection. These studies have been approved by an ethics committee (No 50/2017, Landesamt für Verbraucherschutz, Saarbrücken, Germany). The compounds were orally administered (one rat per compound, n = 5) in an aqueous suspension via gastric intubation in doses of 2 mg/kg body weight (BW). After administration, rats were housed in metabolism cages for 24 h. Rats had water ad libitum during the collection of urine, which was collected separately from feces using metabolism cages. No compounds were administered to the sixth rat, and its urine was used as control.

### 2.2. Sample Preparations

#### 2.2.1. Urine Conjugate Cleavage for the Identification of Phase I Metabolites by LC-HRMS/MS

According to a previously published procedure, with minor modifications [13], 2 mL of rat urine (adjusted to pH 5.2 with acetic acid, 1 M, approximately 50 µL) was incubated at 56 °C for 3 h with 50 µL of a glucuronidase (EC no. 3.2.1.31, Merck) and arylsulfatase (EC no. 3.1.6.1, Merck) mixture from *Helix pomatia*. This procedure was performed in duplicate per rat (twelve samples in total); one aliquot of cleaved urine samples (UGLUC) was extracted via liquid–liquid extraction (LLE) and the second via solid-phase extraction (SPE).

#### 2.2.2. Urine LLE for the Identification of Phase I Metabolites by LC-HRMS/MS

LLE was performed as described elsewhere, with minor modifications [14]. A volume of 2 mL of UGLUC was acidified using 1 mL of HCl (37%), and then 2 mL of ammonium sulfate solution (30%) and 1 mL of NaOH solution (10 M) were added (final pH 8–9). This mixture was extracted using 5 mL of ethyl acetate/DCM/isopropanol (3:1:1, *v*/*v*/*v*), followed by manually shaking for 20 s and centrifugation for two minutes at 3000× *g*. The organic layer was transferred into a glass flask, evaporated to dryness at 60 °C, and reconstituted with 100 µL of MeOH. A 10 µL aliquot of each sample was injected onto the LC-HRMS/MS system.

#### 2.2.3. Urine SPE for the Identification of Phase I Metabolites by LC-HRMS/MS

As described elsewhere, with minor modifications [13], HCX cartridges were conditioned with 1 mL of MeOH and 1 mL of purified water. The cartridges were loaded with 2 mL of UGLUC and washed with 1 mL of purified water, 1 of mL HCl (0.1 M), and 2 mL of MeOH. Target compounds were eluted using 1 mL of a MeOH/NH_3_ mixture (35%, 98/2, *v*/*v*). The eluates were evaporated to dryness at 70 °C under a gentle stream of nitrogen, and residues were dissolved in 50 µL of MeOH. A 10 µL aliquot of each sample was injected onto the LC-HRMS/MS system.

#### 2.2.4. Urine Precipitation for the Identification of Phase II Metabolites by LC-HRMS/MS

Urine samples were prepared as described elsewhere, with minor modifications [15]. A volume of 0.1 mL of native urine was mixed with 0.5 mL of ACN containing 0.1 mg/mL of diazepam-d_5_. The mixture was shaken in a rotary shaker for 2 min at 2000 rpm, centrifuged at 18,407× *g*, and then 0.5 mL was transferred into a glass vial and evaporated to dryness at 70 °C under a gentle stream of nitrogen. The residue was dissolved in 50 µL of a mixture of 2 mM ammonium formate solution containing 0.1% (*v*/*v*) formic acid (eluent A) and 2 mM ammonium formate solution in acetonitrile/methanol (50:50, *v*/*v*) containing 0.1% (*v*/*v*) formic acid and 1% (*v*/*v*) water (eluent B; 1:1, *v*/*v*) and 10 µL was injected onto the LC-HRMS/MS system.

#### 2.2.5. Urine SPE for the Identification of Phase II Metabolites by LC-HRMS/MS

Native rat urine samples were extracted via SPE as described in Section 2.2.3.

#### 2.2.6. Urine Sample Preparation Prior to GC-MS

Urine samples were prepared according to a previously published procedure, with minor modifications [16,17]. Samples were divided into two aliquots of 2.5 mL, one aliquot was mixed with 0.1 mL of internal standard solution (0.1 mg/mL diazepam-d_5_ in MeOH), and 1 mL of HCl (37%) was added. This mixture was hydrolyzed for 15 min under reflux and 2 mL of ammonium sulfate solution (30%) and 1 mL of NaOH solution (10 M) were added (final pH 8–9). Then, the second aliquot of 2.5 mL of untreated urine was added. This mixture was extracted using 5 mL of ethyl acetate/DCM/isopropanol (3:1:1, *v*/*v*/*v*), followed by manually shaking for 20 s and centrifugation for two minutes at 3000× *g*. The organic layer was transferred into a glass flask and evaporated to dryness at 60 °C. Samples were then derivatized using a mixture of acetic aldehyde and pyridine (3:2, *v*/*v*), coupled with microwave irradiation for 5 min at 580 W. After the acetylation, extracts were evaporated to dryness at 60 °C, reconstituted with 100 µL MeOH, and 1 µL was injected onto the GC-MS system.

### 2.3. Human In Vitro Incubations for the Conformation of In Vivo Rat Phase I Metabolites

According to previously published procedures, with minor modifications [18,19], deschloroketamine derivatives with a final concentration of 25 µM were incubated with pHLMs (1 mg microsomal protein/mL). Additionally, the reaction mixture contained 90 mM phosphate buffer (pH 7.4), superoxide dismutase (200 U/mL), isocitrate (5 mM), MgCl_2_ (5 mM), isocitrate dehydrogenase (0.5 U/mL), and NADP^+^ (1.2 mM). Incubations were performed at 37 °C for 60 min and terminated by adding 50 µL of ice-cold ACN containing 0.1 mg/mL of diazepam-d_5_. The mixtures were then centrifuged at 18,407× *g* for 5 min, before 70 µL of the supernatants was transferred into autosampler vials and 10 µL was injected onto the LC-HRMS/MS system. Negative controls without pHLMs, for every deschloroketamine derivative, and blank incubations containing pHLMs without the deschloroketamine derivatives, were prepared to identify the compounds without a metabolic origin. All incubations were performed in duplicate.

### 2.4. LOI

#### 2.4.1. LOI for Analysis by LC-HRMS/MS

LOI, by LC-HRMS/MS (n = 3), were determined as follows. A volume of 90 µL of blank human urine was fortified with 10 µL of an analyte mixture in MeOH at concentrations of 1000 µg/L, 100 µg/L, 10 µg/L, and 1 µg/L. All given concentrations are the final urine concentrations. Spiked urine samples were extracted via urine precipitations, as described in Section 2.2.4, which corresponds to the LC-HRMS/MS SUSA sample preparation. A 10 µL aliquot each was injected onto the LC-HRMS/MS system. The criteria for the LOI were fulfilled when the S/N was at least 3 and the compound could be identified via its MS^2^ spectra using an extended version of the Maurer/Meyer/Helfer/Weber 2018 library [20].

#### 2.4.2. LOI for Analysis by GC-MS

LOI, for GC-MS (n = 3), were determined as follows. A volume of 4.5 mL of blank human urine was fortified with 500 µL of an analyte mixture in MeOH at concentrations of 20 mg/L, 10 mg/L, 1 mg/L, and 0.1 mg/L. All given concentrations are the final urine concentrations. Spiked urine samples were extracted by LLE after partial urine hydrolysis followed by acetylation (UHyAc), as described in Section 2.2.6, which corresponds to the GC-MS SUSA sample preparation. A 1 µL aliquot of each sample was injected onto the GC-MS system. The criteria for the LOI were fulfilled when the compound could be identified via the automated mass spectral deconvolution and identification system (AMDIS, https://chemdata.nist.gov/dokuwiki/doku.php?id=chemdata:amdis (accessed on 2 January 2024)) in simple mode. Further AMDIS settings were previously described [21] and were used with the following modifications: width, 20; adjacent peak subtraction, 2; resolution, high; sensitivity, very high; shape requirement, low; minimal match factor, 50.

### 2.5. Detectability in Rat Urine Using SUSA

SUSAs were performed as described earlier [14,22,23], including urine precipitation (UPP) in combination with LC-HRMS/MS or UHyAc in combination with GC-MS. Furthermore, the metabolites of the deschloroketamine derivatives detected via both SUSAs were compared to the ketamine metabolites detected in human urine samples submitted to the authors’ clinical toxicology laboratory for regular toxicological analyses.

### 2.6. Human Urine Sample

A urine sample was submitted to the authors’ laboratory for toxicological analysis after a suspected intake of 2-oxo-PCMe. The urine sample was prepared and analyzed following both GC-MS SUSA and LC-HRMS/MS SUSA.

### 2.7. LC-HRMS/MS Apparatus

According to a previous study, with minor changes [24], a Thermo Fisher Scientific (TF, Dreieich, Germany) Dionex UltiMate 3000 consisting of a degasser, a quaternary pump, a DL W2 wash system, and a HCT PAL autosampler (CTC Analytics AG, Zwinger, Switzerland) was used. The system was coupled to a TF Q Exactive orbitrap mass spectrometer, equipped with a heated electrospray ionization II source (HESI-II). According to the manufacturer’s recommendations, calibration was performed prior to analysis using external mass calibration. The conditions of the LC system were as follows: a TF Accucore Phenyl Hexyl column (100 × 2.1 mm ID, 2.6 µm particle size); gradient elution using eluents A and B. The flow rate was set as follows: 0–11.5 min at 0.500 mL/min and 11.5–13.5 min at 0.800 mL/min. The following gradient settings were used: 0–1.0 min hold 1% B, 1–10 min to 99% B, 10–11.5 min hold 99% B, and 11.5–13.5 min hold 1% B. The HESI-II source conditions were as follows: ionization mode, positive; heater temperature, 320 °C; ion transfer capillary temperature, 320 °C; sheath gas, 60 arbitrary units (AU); auxiliary gas, 10 AU; spray voltage, 4.00 kV; and S lens RF level, 50.0. Mass spectrometry experiments for the identification of metabolites in rat urine were performed using a full scan mode and data-dependent acquisition (DDA) with an inclusion list containing the masses of the expected metabolites. For full data scans, the settings were as follows: resolution, 35,000 at *m*/*z* 200; automatic gain control (AGC) target, 1e6; maximum injection time (IT), 120 ms; and scan ranges: *m*/*z* 130–530. The settings for the DDA with the respective inclusion lists were as follows: resolution, 17,000 at *m*/*z* 200; AGC target, 2e5; maximum IT, 250 ms; loop count, 5; isolation window, 1.0 *m*/*z*; stepped normalized collision energy, 17.5, 35, 52.5%; and pick others, enabled. The HESI-II source settings using SUSA conditions were the same as for the identification of metabolites, with one difference: the ionization mode was set to positive and negative. The mass spectrometry settings using SUSA conditions for a full data scan were as follows: resolution, 35,000 at *m*/*z* 200; automatic gain control (AGC) target, 1e6; maximum injection time (IT), 120 ms; and scan ranges: *m*/*z* 130–930. The settings for the DDA without the inclusion list were as follows: resolution, 17,000 at *m*/*z* 200; AGC target, 2e5; maximum IT, 250 ms; loop count, 5; isolation window, 1.0 *m*/*z*; and stepped normalized collision energy, 17.5, 35, 52.5%. ChemSketch 2018 2.1 (ACD/Labs, Toronto, Canada) was used to draw the structures of the hypothetical metabolites and for the calculations of their exact masses. Xcalibur Qual Browser version 4.1.31.9 (TF, Dreieich, Germany) was used for data handling.

### 2.8. GC-MS Apparatus

According to a previously published procedure, with minor modifications [14], a Hewlett Packard (HP, Agilent Technologies, Waldbronn, Germany) 5890 II gas chromatograph combined with a HP 5972 MSD mass spectrometer and a HP MS ChemStation (DOS series), with HP G1034C software version C03.00, was used for systematic urine screening. The condition of the GC system were as follows: Macherey Nagel (Düren, Germany) capillary dimethylpolysiloxane columns (Optima 1 MS; 12 m × 0.2 mm I. D.; film thickness, 0.35 μm); splitless injection mode; column and injector port temperature, 280 °C; carrier gas, helium; flow rate, 1 mL/min; column temperature, programmed from 90 to 310 °C at 30 °C/min—initial time 2 min, final time 5 min. The MS conditions were as follows: full scan mode, *m*/*z* 50–550; electron ionization (EI) mode; ionization energy, 10 eV; ion source temperature, 150 °C; capillary direct interface, heated to 280 °C. Data Analysis standalone (G1034C Version C.03.00, Hewlett Packard) was used for the data evaluation of the samples analyzed for the detection of metabolites in rat urine. Data obtained by GC-MS using SUSA conditions were evaluated using AMDIS; the settings were as described in Section 2.4.2. An extended version of the Maurer/Meyer/Pfleger/Weber 2023 library was used as the target library [14].

## 3. Results

### 3.1. Identification of In Vivo Phase I Metabolites Using LC-HRMS/MS

All phase I metabolites of 2-oxo-PCcP, 2-oxo-PCE, 2-oxo-PCiP, 2-oxo-PCMe, and 2-oxo-PCPr identified in rat urine after their oral administration are listed in Tables S1–S5, and their mass spectra are shown in Figures S1–S5 in the electronic Supplementary Materials (ESMs). The metabolite ID, masses of precursor ions (PIs) and characteristic fragment ions (FIs) in the MS^2^ spectra, calculated exact masses, elemental compositions, deviation of the measured masses from the calculated masses in parts per million (ppm), and retention times (RTs) are included in these tables and/or figures. Three phase I metabolites of 2-oxo-PCcP, four of 2-oxo-PCE, seven of 2-oxo-PCiP, six of 2-oxo-PCMe, and nine of 2-oxo-PCPr were tentatively identified. The masses of their PIs and FIs described below are calculated exact masses.

#### 3.1.1. Higher-Energy Collisional Dissociation (HCD) Fragmentation Patterns of 2-Oxo-PCcP and Its Phase I Metabolites

The MS^2^ of 2-oxo-PCcP (PI at *m*/*z* 230.1539) showed an initial loss of water (−18.0106 u) leading to FI at *m*/*z* 212.1434 or a loss of cyclopropylamine (−57.0578 u) forming FI at *m*/*z* 173.0961, which corresponds to the 2-oxo-phenylcyclohexyl core structure. This core structure was fragmented by several neutral losses: CO, resulting in an FI at *m*/*z* 145.1012; ethanone (FI at *m*/*z* 129.0699); propanone (FI at *m*/*z* 117.0699); and pentanone (FI at *m*/*z* 91.0542). This is in accordance with the fragmentation pattern of 2-oxo-PCE described by Larabi et al. [11]. Furthermore, the FI at *m*/*z* 58.0651 was detected as corresponding to the mass of protonated cyclopropylamine. CM1 (*N*-dealkylation, PI at *m*/*z* 190.1226) showed an initial neutral loss of ammonia (−17.0265 u) leading to an FI at *m*/*z* 173.0961. Further FIs at *m*/*z* 145.1012, 129.0699, 117.0699, and 91.0542 (the most abundant) corresponded with the fragmentation pattern of the parent compound. The FI at *m*/*z* 67.0542 resulted from the cleavage of the cyclohexanone ring, followed by a neutral loss of CO and the formation of a cyclopentene cation. Two metabolites with PIs at *m*/*z* 246.1489 (hydroxylation isomers 1 and 2) were detected as well, showing mass shifts of +15.9949 u compared to their parent compound. Hydroxylation isomer 1 (CM3) showed an initial loss of cyclopropylamine (−57.0578 u), which led to an FI at *m*/*z* 189.0910. The hydroxy group was localized at the cyclohexanone ring due to the mass shift of −2.0157 u of the FI at *m*/*z* 171.0804 compared to the FI at *m*/*z* 173.0961 in the MS^2^ of the parent compound, as well as the mass shift of +18.0106 u compared to the FI at *m*/*z* 189.0910. Moreover, the FI at *m*/*z* 91.0542 indicated that there was no modification at the benzene ring. CM4’s hydroxy group was localized at the benzene ring due to the mass shift of +15.9949 u between the FIs at *m*/*z* 161.0961 in the spectrum of CM4 and 145.1012 in the parent spectrum, and the FIs at *m*/*z* 107.0491 (CM4) and 91.0542 (parent compound).

#### 3.1.2. HCD Fragmentation Patterns of 2-Oxo-PCE and Its Phase I Metabolites

The MS^2^ of 2-oxo-PCE (PI at *m*/*z* 218.1539) showed an initial loss of ethylamine (−45.0578 u), forming an FI at *m*/*z* 173.0961, which corresponds to the 2-oxo-phenylcyclohexyl core structure, or a loss of water resulting in an FI at *m*/*z* 200.1434. The core structure fragmented as described for 2-oxo-PCcP, including an FI at *m*/*z* 67.0542 (cyclopentene cation). The metabolite EM2 was tentatively identified as hydroxylation at the cyclohexanone moiety, with further oxidation to a ketone function, with a PI at *m*/*z* 232.1332. The initial fragmentation step was a neutral loss of ethylamine (FI at *m*/*z* 187.0754) followed by a loss of CO leading to an FI at *m*/*z* 159.0804. Both FIs showed a mass shift of +13.9792 u compared to the corresponding FI in the parent compound’s spectrum; this was achieved by the addition of oxygen (+15.9949 u) and a formal dehydration of the ketone function (−2.0157 u). Furthermore, EM3 and EM4 were identified as isomers of the hydroxylation at the cyclohexanone ring, with almost identical MS^2^ spectra but different retention times. The FIs at *m*/*z* 161.0961 and 189.0910 showed mass shifts of +15.9949 u compared to the corresponding FI in the spectrum of 2-oxo-PCE and +2.0157 u compared to the FI in the spectrum of EM2. The presence of an FI at *m*/*z* 91.0542 in the spectra of EM3 and EM4 ruled out changes at the benzene ring. Moreover, a mass shift of 18.0106 u between the PI at *m*/*z* 234.1489 and the FI at *m*/*z* 216.1383 in both spectra indicated a neutral loss of water.

#### 3.1.3. HCD Fragmentation Patterns of 2-Oxo-PCiP and Its Phase I Metabolites

The MS^2^ of 2-oxo-PCiP (PI at *m*/*z* 232.1696) showed initial losses of either isopropylamine (−59.0735 u), followed by the FI described for 2-oxo-PCcP, corresponding to the fragmentation pattern of the 2-oxo-phenylcyclohexyl core structure, or water, leading to an FI at *m*/*z* 214.1590. Furthermore, a protonated isopropylamine (FI at *m*/*z* 60.0808) was detected. IM2 (*N*-dealkylation + hydroxylation) with a PI at *m*/*z* 206.1176 was identified as follows: the mass shift between the PI and FI at *m*/*z* 189.0910 corresponded to a loss of ammonia (−17.0265 u) and showed a mass shift of +15.9949 u compared to the deamino FI at *m*/*z* 173.0961 in the 2-oxo-PCiP’s MS^2^ spectrum. There were three metabolites (IM4–6) with a PI at *m*/*z* 248.1645 that were identified as products of hydroxylation. Hydroxylation isomer 1 (IM4) was identified as hydroxylation at the cyclohexanone part, as described for EM3 and 4. IM5 was identified as follows: the FI at *m*/*z* 189.0910 showed a hydroxylation at the 2-oxo-phenylcyclohexyl core due to a mass shift of +15.9949, compared to the FI at *m*/*z* 173.0961 in the parent compound’s MS^2^ spectrum; the loss of CO led to an FI at *m*/*z* 161.0961. Furthermore, the FI at *m*/*z* 107.0491 indicated a hydroxylation of the phenyl part, however, the exact position could not be determined. The hydroxyl group of IM6 was located at the isopropyl structure. The spectrum of IM6 showed an initial loss of water (−18.0106 u) leading to an FI at *m*/*z* 230.1539, followed by a loss of 2-propenamine (FI at *m*/*z* 173.0961) and a loss of CO (FI at *m*/*z* 145.1012) or ethanone (FI at *m*/*z* 129.0699). Furthermore, the FI at 91.0542 showed that the hydroxylation was not located at the phenyl part, the whole FIs at *m*/*z* 76.0757 (C_3_H_9_NO) and 58.0651 (C_3_H_7_N) indicated that the hydroxyl group’s location was in the isopropyl part.

#### 3.1.4. HCD Fragmentation Patterns of 2-Oxo-PCMe and Its Phase I Metabolites

The MS^2^ of 2-oxo-PCMe (PI at *m*/*z* 204.1383) showed an initial loss of methylamine (−32.0422 u, FI at *m*/*z* 173.0961) or water (−18.0106 u, FI at *m*/*z* 186.1277), followed by the fragmentation pattern of the 2-oxo-phenylcyclohexyl core structure described for 2-oxo-PCcP. Metabolite MM3 was identified as a *N*-dealkylation followed by hydroxylation to a hydroxylamine, with a retention time of 5.61 min compared to 5.0 min (*N*-dealkylated metabolite MM1). The initial fragmentation step was a loss of water (−18.0106 u, FI at *m*/*z* 188.1070), followed by the fragmentation pattern of the 2-oxo-phenylcyclohexyl core structure, including the following FIs at *m*/*z* 173.0961, 145.1012, 129.0699, and 91.0542. Two hydroxylation metabolites were detected with PIs at *m*/*z* 220.1332: MM5 was identified as a hydroxylation at the cyclohexanone part, and MM6 as a hydroxylation at the phenyl part. Their identification was performed as described above. In brief: MM5 showed an initial loss of water leading to an FI at *m*/*z* 202.1226 and an initial loss of methylamine (FI at *m*/*z* 189.0910), followed by a further loss of water (FI at *m*/*z* 171.0804) and CO (FI at *m*/*z* 143.0855). In the MS^2^ of MM6, the FI at *m*/*z* 189.0910 resulted from an initial loss of methylamine, followed by a loss of CO (FI at *m*/*z* 161.0961), with further fragmentation to an FI at *m*/*z* 107.0491 (C_7_H_7_O) and an FI at *m*/*z* 67.0542.

#### 3.1.5. HCD Fragmentation Patterns of 2-Oxo-PCPr and Its Phase I Metabolites

The MS^2^ of 2-oxo-PCPr (PI at *m*/*z* 232.1696) showed an initial loss of water (−18.0106 u, FI at *m*/*z* 214.1590) or propylamine (−59.0735 u, FI at *m*/*z* 173.0961), followed by the fragmentation pattern of the 2-oxo-phenylcyclohexyl core structure described for 2-oxo-PCcP. There were two metabolites with PIs at *m*/*z* 262.1438: PM6 was identified as a metabolite obtained via dihydroxylation and the further oxidation of one hydroxy group to a ketone. PM6 showed initial neutral loss of water (−18.0106 u) resulting in an FI at *m*/*z* 244.1332. A second initial fragmentation step starting at the PI was the loss of 3-hydroxypropylamine (−75.0684 u) leading to an FI at *m*/*z* 187.0754; the further loss of CO resulted in an FI at *m*/*z* 159.0804. Both FIs were also detected in the spectrum of the ketone metabolite EM2 of 2-oxo-PCE. Finally, an FI at *m*/*z* 76.0757 (C_3_H_10_ON) was also detected in this spectrum and identified as the protonated 3-hydroxypropylamine group. PM7 was identified as a hydroxylation with further oxidation to a carboxylic acid, also showing an initial loss of water (FI at *m*/*z* 244.1332). The loss of 3-aminopropanoic acid led to an FI at *m*/*z* 173.0961 (2-oxo-phenylcyclohexyl core) and fragmentation as already described before. Furthermore, there were two metabolites with PIs at *m*/*z* 264.1594, identified as dihydroxylation isomers (PM8, PM9). PM8 was identified as follows: two initial losses of water (−18.0106 u each), with FIs at *m*/*z* 246.1489 and 228.1383 and an FI at *m*/*z* 189.0910 resulting from the neutral loss of 3-hydroxypropylamine (−75.0684 u) as a further possible initial fragmentation step. The FIs at *m*/*z* 189.0910, 171.0804, and 143.0855 showed that one hydroxy group was located at the cyclohexanone ring, while the second hydroxy group was identified at the propyl group, due to an FI at *m*/*z* 76.0757 (C_3_H_10_ON). In the MS^2^ of PM9 there was also a mass shift of −18.0106 u between the PI and FI at *m*/*z* 246.1489, indicating a neutral loss of water. Furthermore, an FI at *m*/*z* 76.0757 was detected, which meant that one hydroxy group was located at the propyl chain. The FIs at *m*/*z* 189.0910, 161.0961, and 107.0491 allowed for the localization of the second hydroxy group at the phenyl ring.

### 3.2. Identification of In Vivo Phase II Metabolites Using LC-HRMS/MS

The phase II metabolites of 2-oxo-PCcP, 2-oxo-PCE, 2-oxo-PCiP, 2-oxo-PCMe, and 2-oxo-PCPr identified in rat urine after their oral administration are listed in Tables S1–S5, and their mass spectra are shown in Figures S1–S5 in the ESMs. The metabolite ID, masses of PIs and characteristic FIs in MS^2^, calculated exact masses, elemental compositions, deviation of the measured from the calculated masses in ppm, and RTs are included in these tables and/or figures. Two phase II metabolites of 2-oxo-PCcP, one of 2-oxo-PCE, two of 2-oxo-PCiP, three of 2-oxo-PCMe, and two of 2-oxo-PCPr were tentatively identified. The masses of PIs and FIs described below are calculated exact masses.

#### 3.2.1. HCD Fragmentation Patterns of Common Phase II Metabolites

A *N*-dealkylation and glucuronidation was detected as the phase II metabolite of all five deschloroketamine derivatives (CM5, EM5, IM8, MM8, and PM10), with a PI at *m*/*z* 366.1547. It showed a mass shift of +176.0321 u compared to the corresponding phase I metabolite with a PI at *m*/*z* 190.1226. The initial fragmentation steps were two consecutive losses of water (−18.0106 u), leading to FIs at *m*/*z* 348.1442 and 330.1336. Furthermore, FIs at *m*/*z* 173.0961, 145.1012, 129.0699, and 91.0542 were also present in the MS^2^ spectrum of its corresponding phase I metabolite. *N*-Dealkylation followed by acetylation was detected as the phase II metabolite of 2-oxo-PCcP (CM2) and 2-oxo-PCMe (MM7), with the PI at *m*/*z* 232.1332 showing a mass shift of +42.0106 u compared to the corresponding phase I metabolite. The MS^2^ spectrum included the characteristic FIs also detected in the MS^2^ spectrum of the phase I metabolite, including FIs at *m*/*z* 173.0961, 145.1012, 129.0699, and 91.0542.

#### 3.2.2. HCD Fragmentation Patterns of 2-Oxo-PCiP Phase II Metabolites

IM9 was identified as a glucuronide of the hydroxy metabolite isomer 3 of 2-oxo-PCiP, with a PI at *m*/*z* 424.1966 and a mass shift of +176.0321 u compared to the corresponding phase I metabolite (IM6). Characteristic FIs, detected in the MS^2^ spectrum of the phase I metabolite, were detected in the MS^2^ spectrum of the phase II metabolite as well, including FIs at *m*/*z* 230.1539, 173.0961, 145.1012, 129.0699, 91.0542, and 76.0757.

#### 3.2.3. HCD Fragmentation Patterns of 2-Oxo-PCMe Phase II Metabolites

A further phase II metabolite of 2-oxo-PCMe was formed after *N*-dealkylation, combined with hydroxylation and further glucuronidation (MM9), with a PI at *m*/*z* 382.1469. It showed a mass shift of +176.0321 u compared to the corresponding phase I metabolite and its MS^2^ spectrum contained the characteristic FIs which were also detected in the phase I metabolite, including FIs at *m*/*z* 171.0804, 161.0961, 143.0855, and 91.0542. Furthermore, the spectrum showed an initial loss of water −18.0106 u, leading to an FI at *m*/*z* 364.1391.

#### 3.2.4. HCD Fragmentation Patterns of 2-Oxo-PCPr Phase II Metabolites

PM11 (PI at *m*/*z* 424.1966) was identified as a glucuronide of the hydroxylation metabolite isomer 2 of 2-oxo-PCPr (PM4). Its identification was performed as follows: the cleavage of glucuronic acid led to an FI at *m*/*z* 248.1645, which corresponds to the PI of the phase I metabolite. This was followed by either a loss of water (FI at *m*/*z* 230.1539) or cleavage of 3-hydroxypropylamine (FI at *m*/*z* 173.0961) and further fragmentation of the 2-oxo-phenylcyclohexyl core, as described in the phase I metabolite section. Additionally, an FI at *m*/*z* 76.0757 was detected.

### 3.3. Identification of Metabolites Using GC-MS

The parent compounds could not be detected in rat urine using a GC-MS analysis, but the acetylated *N*-dealkylation metabolite of each deschloroketamine derivative was found. The GC-MS spectra are depicted in Figure S6 (ESMs) and more details can be found in Table S6 (ESMs).

#### EI Fragmentation Patterns of Deschloroketamine Derivative and Their Metabolites

The mass spectrum of 2-oxo-PCcP showed an initial loss of CHO^+^, shown by a loss of 29 u between the PI at *m*/*z* 229 and FI at *m*/*z* 200, and the additional cleavage of cyclopropylamine led to an FI at *m*/*z* 145. The combined cleavage of the cyclopropylamine and loss of water led to an FI at *m*/*z* 172. The further loss of C_3_H_5_ resulted in an FI at *m*/*z* 104, while the FI at *m*/*z* 91 corresponded to a benzyl cation. 2-Oxo-PCE displayed a PI at *m*/*z* 217. Cleavage CO led to an FI at *m*/*z* 189 and a subsequent loss of the ethyl group led to an FI at *m*/*z* 160. Further cleavage of the amine led to an FI at *m*/*z* 146, and the cleavage of carbons led to FIs at *m*/*z* 132, 117, 104, and 91. 2-Oxo-PCiP was identified as follows: an initial loss of CO led to a mass shift of −28 u between the PI at *m*/*z* 231 and FI at *m*/*z* 203. An FI at *m*/*z* 174 resulted from the cleavage of isopropylamine, while a loss of CO in combination with isopropyl chain cleavage led to an FI at *m*/*z* 160. The further loss of carbons led to FIs at *m*/*z* 132, 117, 104, and 91. Only 2-oxo-PCMe AC, with a PI at *m*/*z* 245, could be identified. An initial loss of CO led to an FI at *m*/*z* 217, the initial loss of the amine group resulted in an FI at *m*/*z* 174, with further fragmentation of the cyclohexanone part leading to FIs at *m*/*z* 144, 132, 118, 104, and 91. 2-Oxo-PCPr showed a similar mass spectrum compared to 2-oxo-PCiP, with a PI at *m*/*z* 231 and the initial cleavage of CO resulting in an FI at *m*/*z* 203. Further FIs at *m*/*z* 174, 160, 144, 132, 104, and 91 were also detected. Furthermore, 2-oxo-PCPr AC could be identified, with a PI at *m*/*z* 273. The initial fragmentation step was a loss of C_2_H_4_, leading to an FI at *m*/*z* 245. FIs at *m*/*z* 203, 174. 160, 132, 117, 104, and 91 were detected as described for 2-oxo-PCPr. Only the acetylated *N*-dealkylation metabolite could be detected for all five deschloroketamine derivatives. Its identification was performed as follows: a mass shift of −43 u between the PI at *m*/*z* 231 and FI at *m*/*z* 188 indicated the loss of an acetyl group, the FI at *m*/*z* 174 corresponded to a cleavage of the amine function, a further loss of CH_2_O (−30 u) at the cyclohexanone core led to an FI at *m*/*z* 144, a loss of C_4_H_6_O to an FI at *m*/*z* 104, and a loss of CH_2_ to an FI at *m*/*z* 91. The FI at *m*/*z* 132 resulted from the cleavage of the amine group combined with the subsequent loss of C_2_H_2_O.

### 3.4. Confirmation of In Vivo Phase I Metabolites Using Human In Vitro Incubations

In vitro pHLM incubations confirmed 19 of the 29 phase I metabolites identified in vivo. The metabolic systems in which the metabolites were identified are given in Tables S1–S5 (ESMs). Monohydroxylation and *N*-dealkylation metabolites, as well as a metabolite formed by a combination of both metabolic reactions, were identified in vivo and in vitro. Further oxidated metabolites such as ketones, dihydroxylations, and carboxylic acid were not detected after human in vitro incubations.

### 3.5. Proposed In Vivo Metabolic Pathways

**Figure 2:**
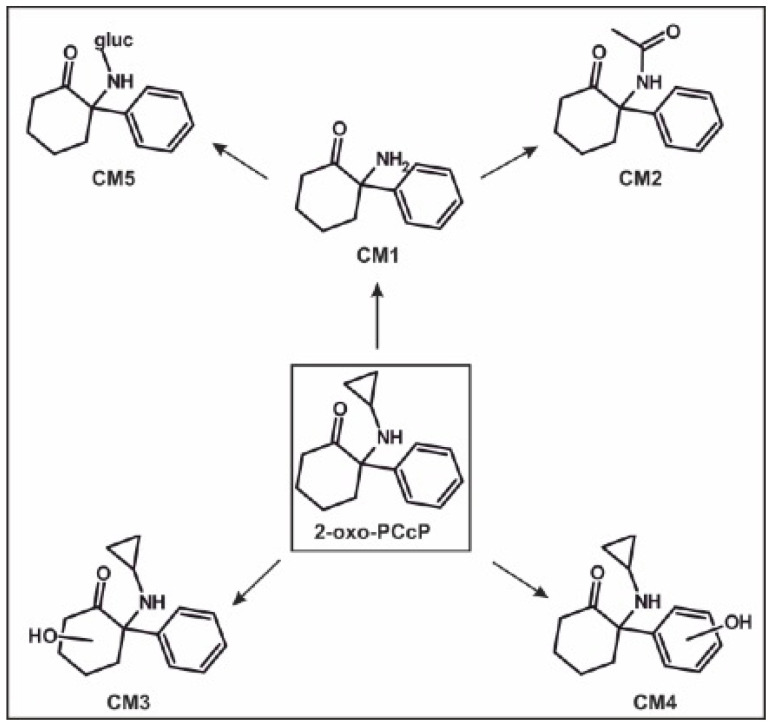
In vivo metabolic pathways of 2-oxo-PCcP. ID corresponding to Table S1. CM, 2-oxo-PCcP metabolite; →, metabolized to.

**Figure 3:**
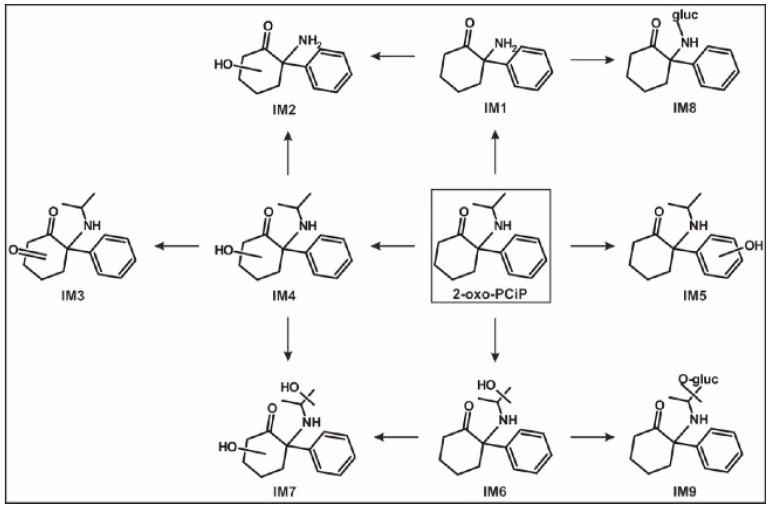
In vivo metabolic pathways of 2-oxo-PCiP. ID corresponding to Table S3. IM, 2-oxo-PCiP metabolite; →, metabolized to.

**Figure 4:**
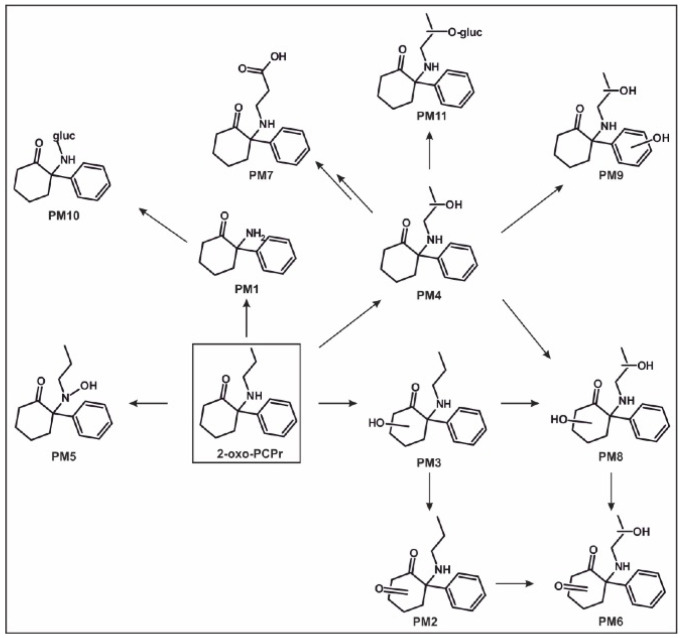
In vivo metabolic pathways of 2-oxo-PCPr. ID corresponding to Table S5. PM, 2-oxo-PCPr metabolite; →, metabolized to.

Based on the identified metabolites in rat urine after oral administrations, the proposed in vivo metabolic pathways of 2-oxo-PCcP, 2-oxo-PCiP, and 2-oxo-PCPr are given in Figure 2, Figure 3 and Figure 4, the pathways of 2-oxo-PCE and 2-oxo-PCMe are given in Figures S7 and S8 (ESMs), and their IDs are given in Tables S1–S5 (ESM).

#### 3.5.1. 2-Oxo-PCcP

*N*-dealkylation led to the formation of CM1. Two different hydroxylation isomers were detected (CM3 and CM4). CM3 was formed by the hydroxylation of the cyclohexanone part, CM4 via the hydroxylation of the phenyl ring. Furthermore, two phase II metabolites were identified, namely, *N*-dealkylation in combination with *N*-acetylation (CM2) or glucuronidation (CM5).

#### 3.5.2. 2-Oxo-PCE

EM1 was formed by *N*-dealkylation. Furthermore, there were two hydroxylation metabolites (EM3 and EM4), both formed via the hydroxylation of the cyclohexanone part. As the exact position of the hydroxy groups could not be determined, both isomers could only be differentiated by their RTs (4.11 min for EM3 and 4.62 for EM4). Further oxidation of the hydroxy group to a ketone led to EM2. There was also one phase II metabolite identified as a *N*-dealkylation, followed by a glucuronidation (EM5).

#### 3.5.3. 2-Oxo-PCiP

The *N*-Dealkylation of the parent compound led to IM1. This was followed by either hydroxylation at the cyclohexanone part (IM2) or glucuronidation (IM8). Three hydroxylation isomers were identified, with IM4 hydroxylated at the cyclohexanone part, IM5 at the phenyl ring, and IM6 at the isopropyl part. The further oxidation of IM4 to the ketone that formed IM3, while a combination of IM4 and IM6 formed dihydroxy metabolite IM7. The glucuronidation of IM6 formed phase II metabolite IM9.

#### 3.5.4. 2-Oxo-PCMe

MM1 was formed by *N*-dealkylation. The further hydroxylation of MM1 led to two isomers, MM2 and MM3, with MM2 hydroxylated at the cyclohexanone part and MM3 formed by *N*-hydroxylation and also showing a higher RT compared to MM1. There were also two hydroxylated metabolites of the parent compound MM5 (hydroxylation at the cyclohexanone part) and MM6 (hydroxylation at the phenyl ring). The further oxidation of MM5 to a ketone resulted in MM4. Their phase II metabolites included the combination of *N*-dealkylation with either *N*-acetylation (MM7) or glucuronidation (MM8) and the glucuronidated *N*-dealkylation + hydroxylation metabolite (MM9).

#### 3.5.5. 2-Oxo-PCPr

The *N*-Dealkylation of 2-oxo-PCPr led to PM1. Hydroxylation of the cyclohexanone part led to PM3, hydroxylation of the propyl chain led to PM4, and *N*-hydroxylation led to the formation of a hydroxylamine (PM5). The further oxidation of PM3 led to PM2 (ketone). PM7 was identified as product of hydroxylation at the propyl chain (PM4) followed by oxidation to a carboxylic acid. Hence, the hydroxy group of PM4 should be located at the terminal carbon in the chain. Furthermore, there were two dihydroxylation isomers (PM8 and PM9), both with a hydroxylation at the propyl chain. PM8 was further hydroxylated at the cyclohexanone ring and PM9 at the phenyl ring. The oxidation of the hydroxy group at the cyclohexanone ring to a ketone of PM8 led to PM6. There were two phase II metabolites identified for 2-oxo-PCPr. PM10 was formed by the glucuronidation of PM1 and PM11 by the glucuronidation of PM4.

### 3.6. LOI in Spiked Urine

The analysis of spiked human urines showed LOI at levels of 10 µg/L after urine precipitation, followed by their analysis via LC-HRMS/MS. Using UHyAc in combination with GC-MS, the LOI were determined to be at 1 mg/L for all parent compounds except 2-oxo-PCMe (acetylated) and 2-oxo-PCcP (LOI at 10 mg/L).

### 3.7. Comparison of Different Sample Preparations and Their Detectability in Rat Urine

Tables S7–S11 (ESM) show the metabolites detected using LC-HRMS/MS and GC-MS in combination with different sample preparations. The analysis using SPE in combination with LC-HRMS/MS enabled the detection of all parent compounds and all metabolites, except two (IM5 and IM6). The UGLUC SPE in combination with LC-HRMS/MS led to the detection of 32 out of the 44 possible metabolites and parent compounds; 31 hits were achieved using UGLUC LLE, and 29 using UPP. The least number of metabolites was detectable via GC-MS in combination with UHyAc, where only the *N*-dealkylation metabolites could be identified.

### 3.8. Toxicological Detectability Using SUSA

Table 1 shows the parent compounds and metabolites detected in rat urine using the LC-HRMS/MS SUSA. 2-Oxo-PCE, 2-oxo-PCMe, 2-oxo-PCPr, *N*-dealkylation metabolites (CM1, EM1, IM1, MM1, and PM1), and *N*-dealkylations in combination with glucuronidation (CM5, EM5, IM9, MM8, and PM10) were detectable. Furthermore, hydroxylation metabolites (EM3, PM8, and PM9) and further oxidized metabolites (EM2, IM3, PM2, PM6, and PM7) were detected. The comparison of their absolute peak areas showed that CM5, EM5, IM8, MM8 (each *N*-dealkylation + glucuronidation metabolites), and PM7 (hydroxylation isomer 2 + oxidation to carboxylic acid) were the most abundant metabolites. Out of these five metabolites, only PM7 was a compound-specific metabolite. Further compound-specific metabolites with high absolute peak areas were EM2 and IM3. Regarding 2-oxo-PCMe, the parent compound itself showed the highest absolute peak area when using the LC-HRMS/MS SUSA. No compound-specific metabolites could be detected in these experiments for 2-oxo-PCcP. Table 2 shows the metabolites detected in rat urine using the GC-MS SUSA. Here, only acetylated *N*-dealkylation metabolites were detectable. Metabolic yields were calculated using the respective metabolites’ absolute peak areas.

### 3.9. Analysis of a Human Urine Sample

The analysis of a human urine sample after UPP in combination with LC-HRMS/MS allowed for the identification of 2-oxo-PCMe and several phase I metabolites, including a *N*-dealkylation (MM1), reduction (not identified in rat urine in this study), hydroxylation (MM5 and MM6), and hydroxylation with further oxidation to a ketone (MM4), while no phase II metabolites could be detected. A screening using GC-MS after UHyAc only allowed for the detection of MM1. The most abundant analytical targets were MM1 and 2-oxo-PCMe.

## 4. Discussion

Larabi et al. investigated the in vitro metabolism of 2-oxo-PCE after an HLM incubation and identified 12 phase I and 3 phase II metabolites [11]. This included *N*-dealkylation, hydroxylation, reduction, oxidative deamination, and dehydration metabolites in phase I and *N*- as well as *O*-glucuronides as phase II metabolites. A comparison with its metabolism in rat showed predominant differences concerning reduction and dehydration. Furthermore, no *O*-glucuronide metabolites of 2-oxo-PCE could be detected in rat urine, whereas two hydroxylation isomers were detected in this study, compared to one detected by Larabi et al. Differences in metabolite detection might be explained due to use of different systems and species (in vivo rat vs. in vitro HLM) and different concentrations of parent compounds. The metabolism of 2-oxo-PCMe in rats (urine, serum, and brain tissue) after the subcutaneous administration of 30 or 10 mg/kg BW was investigated by Hájková et al. [10]. The authors identified 17 phase I and 9 phase II metabolites, including *N*-dealkylation, hydroxylation, and reduction of the cyclohexanone metabolites, as well as combinations of these transformations in phase I and glucuronides in phase II metabolism. The differences in the number and type of metabolites detected compared to the study by Hájková et al. might reflect differences in our doses and/or route of administration, as well as the different biosamples obtained (serum and brain tissue in addition to urine samples).

The differences between the metabolites detected in the in vivo and in vitro experiments were related to further oxidized metabolites. This could be due to the 24 h time period of the rat experiments (compared to the 1 h for the pHLM incubations) allowing for further metabolic steps, which is a known challenge when comparing in vivo with in vitro models [25,26].

The analysis of a human urine sample after a suspected intake of an unknown amount of 2-oxo-PCMe showed similar results for the phase I metabolites as the metabolites detected in rat urine, while no phase II metabolites could be detected in the human urine sample. A noticeable difference was in the detection of a reduced metabolite in human urine; in HLMs by Larabi et al.; and in rat urine, serum, and brain tissue by Hájková et al. This difference might be due to species and/or dosage differences, but also due to different routes of administration compared to the present study. Users of 2-oxo-PCMe reported, in an online forum, doses between 0.3 and 1.5 mg/kg BW and varying routes of administration such as oral, sublingual, intramuscular, or rectal, as well as vaporization or insufflation [27]. So far, no metabolism data on 2-oxo-PCcP, 2-oxo-PCiP or 2-oxo-PCPr are available, nor have reports of their use in online forums, or seizures, been reported by relevant authorities. However, the 3-methoxy-analogs of 2-oxo-PCPr (MXPr) and 2-oxo-PCiP (MxiPr) have been reported as SPDs [28,29].

Using GC-MS, only the acetylated *N*-dealkylation metabolite was detected. Due to the acetylation step during sample preparation, those were largely derived from the phase I metabolites CM1, EM1, IM1, MM1, and PM1, instead of being phase II metabolites. This assumption is supported by the fact that no acetylated *N*-dealkylation metabolites of 2-oxo-PCE, 2-oxo-PCiP, or 2-oxo-PCPr could be detected using the LC-HRMS/MS-based analysis.

In human urine samples collected following a ketamine administration, ketamine, as well as its *N*-dealkylation, hydroxylation, and *N*-dealkylation metabolites, in combination with hydroxylation metabolites, was also frequently detected using the GC-MS-based SUSA [14,15,20]. The detection of the parent compound might be explained by the comparable high doses used for anesthesia (1–4.5 mg/kg BW i.v., 6.5–13 mg/kg BW i.m.) [30].

The current data showed similar metabolic pathways amongst the five deschloroketamine derivatives. *N*-Dealkylation metabolites were the most abundant metabolites detected in rat urine, but they were not compound-specific as they can be formed via the *N*-dealkylation of any derivative with an *N*-substituent. Based on the absolute peak areas of the parent compounds and metabolites detected in the LC-HRMS/MS SUSA EM2, IM3, 2-oxo-PCMe itself, and PM7, might be recommended as specific screening targets. As for 2-oxo-PCcP, no specific metabolites could be detected in the SUSA; only the unspecific metabolites CM1 and CM5 might be used as targets. However, the detection of unspecific metabolites may at least indicate the ingestion of a deschloroketamine derivative. Subsequent analysis using more sensitive approaches, such as SPE prior to an LC-HRMS/MS or GC-MS analysis, might be used for substance-specific detection. Sample preparation by SPE would concentrate the samples, enabling the detection of low-concentrated analytes which were not detectable by both SUSA approaches.

Comparing the different sample preparations and MS approaches used in this study, SPE in combination with LC-HRMS/MS and an inclusion list led to the detection of most metabolites. UGLUC followed by LLE or SPE was used for the identification of phase I metabolites, since the cleavage of phase II metabolites should enhance the detectability of their corresponding phase I metabolites. Extraction via SPE should enhance detectability even more by concentrating samples. SPE without prior conjugate cleavage and UPP were used for the identification of phase II metabolites, as SPE could enhance detectability of low-concentration metabolites as described above. Furthermore, UPP was used as it is a fast and simple sample preparation, and one also used in the LC-HRMS/MS SUSA. For detectability studies, the laboratories’ SUSAs were used (UPP followed by LC-HRMS/MS analysis and UHyAc followed by GC-MS analysis) as they are fast and simple workflows well established in the context of clinical toxicology [14,20]. Of the two screening (SUSA) methods, the LC-HRMS/MS SUSA showed lower LOI compared to the GC-MS SUSA and enabled the detection of substance-specific metabolites, including the metabolites and parent compounds also detected after pHLM incubations. Concerning the LOI, Gomila et al. reported limits for the structurally related 3-methoxy-phenylcyclohexylpiperidine (3-MeO-PCP) using a GC-MS-based approach at concentrations of 1 µg/L in blood and urine [31]. Differences in sensitivity might be explained by different sample preparations, as Gomila et al. used an SPE-based extraction method for their study, in contrast to the LLE in our study. However, the data obtained using GC-MS also allowed for the detection of a deschloroketamine metabolite, as well as of parent compounds in concentrations of at least 1 mg/L.

## 5. Conclusions

In total, 39 metabolites derived from five deschloroketamine derivatives were tentatively identified in rat urine, after their oral administration, by means of LC-HRMS/MS. Human in vitro incubations using pHLMs confirmed 19 phase I metabolites. The most abundant metabolites were formed by *N*-dealkylation, which did not allow for differentiation between the deschloroketamine derivatives only differing in their *N*-substitution. The comparison of different sample preparation strategies showed that UPP followed by an LC-HRMS/MS analysis is sufficient for the detection of specific metabolites for every NPS included in this study, except for 2-oxo-PCcP. In the samples analyzed using GC-MS, only the acetylated *N*-dealkylation metabolites were detected. The LOI were determined at levels of 10 µg/L using UPP prior to LC-HRMS/MS and at 1 mg/L using UHyAc prior to GC-MS, except for 2-oxo-PCcP (10 mg/L). Using the LC-HRMS/MS SUSA, EM2, IM3, 2-oxo-PCMe itself, and PM7 were identified as specific screening targets. Since only the *N*-dealkylated metabolites of 2-oxo-PCiP could be detected in rat urine using the SUSA conditions, no substance-specific screening targets could be suggested for this analyte. A human urine sample after a suspected intake of 2-oxo-PCMe was analyzed as well, and the results were in accordance with those obtained after the analysis of rat urine. The results of this study showed the importance of identifying specific screening targets for structurally similar compounds. Furthermore, all compounds can be detected using different sample preparation procedures combined with LC-HRMS/MS or GC-MS. The data from this study will thus help us to detect deschloroketamine derivatives in the context of forensic and clinical toxicology and for doping control.

## Figures and Tables

**Table 1:** Proposed analytical targets for the LC-HRMS/MS SUSA. PI, precursor ion; CM, 2-oxo-PCcP metabolite; EM, 2-oxo-PCE metabolite; IM, 2-oxo-PCiP metabolite; MM, 2-oxo-PCMe metabolite; PM, 2-oxo-PCPr metabolite; Yield, metabolic yield in %.

Metabolite ID	Metabolic Reaction	Exact Mass of PI, *m*/*z*	RT, min	Absolute Peak Area	Yield, %
CM1	*N*-Dealkylation	230.1539	5.70	4.88 × 10^8^	34
CM5	*N*-Dealkylation + glucuronidation	366.1547	5.35	9.38 × 10^8^	66
2-Oxo-PCE	Parent compound	218.1539	5.44	1.31 × 10^8^	-
EM1	*N*-Dealkylation	190.1226	5.04	6.47 × 10^8^	34
EM2	Hydroxylation + oxidation to a ketone	232.1332	4.75	3.06 × 10^8^	16
EM3	Hydroxylation isomer 1	234.1489	4.11	1.41 × 10^8^	7
EM5	*N*-Dealkylation + glucuronidation	366.1547	5.20	8.21 × 10^8^	43
IM1	*N*-Dealkylation	190.1226	5.03	3.13 × 10^7^	4
IM3	Hydroxylation isomer 1 + oxidation to a ketone	246.1489	5.22	1.92 × 10^8^	27
IM8	*N*-Dealkylation + glucuronidation	366.1547	5.22	4.32 × 10^8^	60
IM9	Hydroxylation isomer 3 + glucuronidation	424.1966	5.24	6.51 × 10^7^	9
2-Oxo-PCMe	Parent compound	204.1483	5.10	1.39 × 10^8^	-
MM1	*N*-Dealkylation	190.1226	5.00	6.86 × 10^8^	36
MM3	*N*-Dealkylation + hydroxylamine	206.1176	5.61	9.25 × 10^6^	1
MM8	*N*-Dealkylation + glucuronidation	366.1547	5.25	1.17 × 10^9^	63
2-Oxo-PCPr	Parent compound	232.1969	5.96	1.90 × 10^7^	-
PM1	*N*-Dealkylation	190.1226	5.05	2.05 × 10^8^	9
PM2	Hydroxylation isomer 1 + oxidation to a ketone	246.1489	5.37	3.68 × 10^8^	14
PM6	Dihydroxylation isomer 1 + oxidation to a ketone	262.1438	4.85	5.67 × 10^7^	2
PM7	Hydroxylation isomer 2 + oxidation to carboxylic acid	262.1438	5.31	8.91 × 10^8^	35
PM8	Dihydroxylation isomer 1	264.1594	4.61	2.65 × 10^7^	1
PM9	Dihydroxylation isomer 2	264.1594	5.09	1.50 × 10^8^	6
PM10	*N*-Dealkylation + glucuronidation	366.1547	5.29	2.33 × 10^8^	9
PM11	Hydroxylation + glucuronidation	424.1966	5.24	6.23 × 10^8^	24

**Table 2:** Proposed analytical targets of five deschloroketamine derivatives for the GC-MS SUSA, including the masses of precursor ions (PIs) and the elemental composition and masses of characteristic fragment ions (FIs). AC, acetylated; RI, retention index.

Metabolite	PI Mass, *m*/*z*	RI	Elemental Composition	Characteristic FI
2-Oxo-PCcP *N*-dealkyl AC	231	1874	C_14_H_17_NO_2_	188, 174, 144, 132, 104, 91
2-Oxo-PCE *N*-dealkyl AC	231	1874	C_14_H_17_NO_2_	188, 174, 144, 132, 104, 91
2-Oxo-PCiP *N*-dealkyl AC	231	1874	C_14_H_17_NO_2_	188, 174, 144, 132, 104, 91
2-Oxo-PCMe *N*-dealkyl AC	231	1874	C_14_H_17_NO_2_	188, 174, 144, 132, 104, 91
2-Oxo-PCPr *N*-dealkyl AC	231	1874	C_14_H_17_NO_2_	188, 174, 144, 132, 104, 91

## Data Availability

The data presented in this study are available on request from the corresponding author. The data are not publicly available due to privacy.

## Supplementary Materials

The following supporting information can be downloaded at https://www.mdpi.com/article/10.3390/metabo14050270/s1, Table S1: List of phase I and II metabolites of 2-oxo-PCcP; Table S2: List of phase I and II metabolites of 2-oxo-PCE; Table S3: List of phase I and II metabolites of 2-oxo-PCiP; Table S4: List of phase I and II metabolites of 2-oxo-PCMe; Table S5: List of phase I and II metabolites of 2-oxo-PCPr; Table S6: Parent compounds and metabolites of five deschloroketamine derivatives detected by GC-MS; Table S7: 2-Oxo-PCcP and its metabolites, detected in rat urine after its oral administration using different sample preparations in combination with LC-HRMS/MS or GC-MS; Table S8: 2-Oxo-PCE and its metabolites, detected in rat urine after its oral administration using different sample preparations in combination with LC-HRMS/MS or GC-MS; Table S9: 2-Oxo-PCiP and its metabolites, detected in rat urine after its oral administration using different sample preparations in combination with LC-HRMS/MS or GC-MS; Table S10: 2-Oxo-PCMe and its metabolites, detected in rat urine after its oral administration using different sample preparations in combination with LC-HRMS/MS or GC-MS; Table S11: 2-Oxo-PCPr and its metabolites, detected in rat urine after its oral administration using different sample preparations in combination with LC-HRMS/MS or GC-MS; Figure S1: HRMS^2^ spectra of 2-oxo-PCcP and its metabolites, detected in rat urine after its oral administration; Figure S2: HRMS^2^ spectra of 2-oxo-PCE and its metabolites, detected in rat urine after its oral administration; Figure S3: HRMS^2^ spectra of 2-oxo-PCiP and its metabolites, detected in rat urine after its oral administration; Figure S4: HRMS^2^ spectra of 2-oxo-PCMe and its metabolites, detected in rat urine after its oral administration; Figure S5: HRMS^2^ spectra of 2-oxo-PCPr and its metabolites, detected in rat urine after its oral administration; Figure S6: EI-MS spectra of 2-oxo-PCcP, 2-oxo-PCE, 2-oxo-PCiP, 2-oxo-PCMe AC, 2-oxo-PCPr, and acetylated *N*-dealkylation metabolites. Figure S7: In vivo metabolic pathways of 2-oxo-PCE; Figure S8: In vivo metabolic pathways of 2-oxo-PCE.

## List of Abbreviations

2-oxo-PCcP, deschloro-N-cyclopropyl-ketamine; 2-oxo-PCE, deschloro-N-ethyl-ketamine; 2-oxo-PCiP, deschloro-N-isopropyl-ketamine; 2-oxo-PCMe, deschloroketamine; 2-oxo-PCPr, deschloro-N-propyl-ketamine; ACN, acetonitrile; AGC, automatic gain control; AUs, arbitrary units; BW, body weight; DCM, dichloromethane; DDA, data-dependent acquisition; EI, electron ionization; EMCDDA, European Monitoring Centre for Drugs and Drug Addiction; ESM, electronic Supplementary Materials; FI, fragment ion; GC-MS, gas chromatography coupled with mass spectrometry; HCl, hydrochloric acid; HCD, higher-energy collisional dissociation; HESI-II, heated electrospray ionization II source; HLMs, human liver microsomes; HP, Hewlett Packard; HPLC, high-performance liquid chromatography; HRMS, high-resolution mass spectrometry; IT, injection time; LC-HRMS/MS, liquid chromatography high-resolution tandem mass spectrometry; LLE, liquid–liquid extraction; LOI, limit(s) of identification; NADP+, nicotinamide adenine dinucleotide phosphate; NaOH, sodium hydroxide; NMR, nuclear magnetic resonance; NPSs, new psychoactive substances; MeOH, methanol; MgCl2, magnesium chloride; MXiPr, methoxisopropamine; MXPr, methoxpropamine; pHLMs, pooled human liver microsomes; PI, precursor ion; ppm, parts per million; RT, retention time; SPE, solid-phase extraction; SUSA, standard urine screening approach; TF, Thermo Fisher Scientific; UGLUC, cleaved urine; UHyAc, partial urine hydrolysis followed by acetylation; UPP, urine precipitation.
