# Supplementary material for: In Vivo and In Vitro Metabolic Fate and Urinary Detectability of Five Deschloroketamine Derivatives Studied by Means of Hyphenated Mass Spectrometry
Source: Metabolites. 2024 May 8;14(5):270. doi: 10.3390/metabo14050270 (PMC11122975; doi:10.3390/metabo14050270)
Supplement: Supplementary file 1 [file metabolites-14-00270-s001.zip › metabolites-2983807-supplementary.pdf]

In Vivo and *in Vitro* Metabolic Fate and Urinary Detectability of Five Deschloroketamine Derivatives Studied by Means of Hyphenated Mass Spectrometry

Fabian Frankenfeld [1], Lea Wagmann [1], Anush Abelian[2], Jason Wallach[2], Adeboye Adejare[2], Simon D. Brandt [3] and Markus R. Meyer [1,*]

[1] Department of Experimental and Clinical Toxicology, Institute of Experimental and Clinical Pharmacology and Toxicology, Center for Molecular Signaling (PZMS), Saarland University, Homburg, Germany; Fabian.Frankenfeld@uks.eu; Lea.Wagmann@uks.eu
[2] Department of Pharmaceutical Sciences, Philadelphia College of Pharmacy, Saint Joseph's University, Philadelphia. Pennsylvania, 19104, USA; aabelian@sju.edu, jwallach@sju.edu, aadejare@sju.edu
[3] School of Pharmacy and Biomolecular Sciences, Liverpool John Moores University, Liverpool L3 3AF, UK; s.brandt@ljmu.ac.uk
* Correspondence: markus.meyer@uks.eu Tel.: +49-6841-16-26438

Keywords: new psychoactive substance; deschloroketamine; deschloro-*N*-ethyl-ketamine; deschloro-*N*-isopropyl-ketamine; deschloro-*N*-cyclopropyl-ketamine; deschloro-*N*-propyl-ketamine; metabolism; *in vivo*; *in vitro*; LC-HRMS/MS;

Table S1. List of *in vivo* phase I and II metabolites of 2-oxo-PCcP, identified in rat urine samples and *in vitro* phase I metabolites identified in incubations using pooled human liver microsomes (pHLM), including the respective metabolite ID, metabolic reaction, masses of the precursor ion (PI) and characteristic fragment ions (FI) detected in MS^2, calculated exact masses, elemental composition, calculated mass errors in parts per million (ppm), retention times (RT) in minutes, and system in which metabolites were identified. The metabolites are sorted by their mass and RT. pHLM, identified in pHLM incubations; rat, identified in rat urine samples

Metabolite ID	Metabolic Reaction	Characteristic Ions Measured Accurate Masses	Calculated Exact Masses, *m/z*	Elemental Composition	Error, ppm	RT, min	Identified in
2-Oxo-PCcP	Parent compound	PI at *m/z* 230.1537	230.1539	$C_{15}H_{20}ON$	-0.91	5.70	pHLM and rat
		FI at *m/z* 212.1430	212.1434	$C_{15}H_{18}N$	-1.68		
		FI at *m/z* 173.0958	173.0961	$C_{12}H_{13}O$	-1.63		
		FI at *m/z* 155.0849	155.0855	$C_{12}H_{11}$	-4.04		
		FI at *m/z* 145.1009	145.1012	$C_{11}H_{13}$	-1.76		
		FI at *m/z* 129.0697	129.0699	$C_{10}H_{9}$	-1.12		
		FI at *m/z* 117.0698	117.0699	$C_{9}H_{9}$	-0.78		
		FI at *m/z* 91.0546	91.0542	$C_{7}H_{7}$	3.72		
		FI at *m/z* 58.0659	58.0651	$C_{3}H_{8}N$	13.0		
CM1	*N*-Dealkylation	PI at *m/z* 190.1222	190.1226	$C_{12}H_{16}ON$	-2.52	5.06	pHLM and rat
		FI at *m/z* 173.0961	173.0961	$C_{12}H_{13}O$	-0.05		
		FI at *m/z* 155.0854	155.0855	$C_{12}H_{11}$	-0.94		
		FI at *m/z* 145.1011	145.1012	$C_{11}H_{13}$	-0.37		
		FI at *m/z* 129.0700	129.0699	$C_{10}H_{9}$	0.89		
		FI at *m/z* 117.0702	117.0699	$C_{9}H_{9}$	2.74		
		FI at *m/z* 91.0548	91.0542	$C_{7}H_{7}$	6.10		
		FI at *m/z* 67.0550	67.0542	$C_{5}H_{7}$	12.0		
CM2	*N*-Dealkylation + acetylation	PI at *m/z* 232.1330	232.1332	$C_{14}H_{18}O_{2}N$	-0.90	6.81	rat
		FI at *m/z* 173.0959	173.0961	$C_{12}H_{13}O$	-1.19		
		FI at *m/z* 145.1015	145.1012	$C_{11}H_{13}$	2.34		
		FI at *m/z* 129.0968	129.0699	$C_{10}H_{9}$	-0.41		
		FI at *m/z* 91.0547	91.0542	$C_{7}H_{7}$	5.47		
		FI at *m/z* 67.0549	67.0542	$C_{5}H_{7}$	10.6		
CM3	Hydroxylation isomer 1	PI at *m/z* 246.1486	246.1489	$C_{15}H_{20}O_{2}N$	-0.95	4.56	pHLM and rat
		FI at *m/z* 189.0907	189.0910	$C_{12}H_{13}O_{2}$	-1.55		
		FI at *m/z* 171.0804	171.0804	$C_{12}H_{11}O$	-0.43		
		FI at *m/z* 143.0856	143.0855	$C_{11}H_{11}$	0.31		
		FI at *m/z* 91.0547	91.0542	$C_{7}H_{7}$	4.89		
		FI at *m/z* 58.0660	58.0651	$C_{3}H_{8}N$	15.0		
CM4	Hydroxylation isomer 2	PI at *m/z* 246.1490	246.1489	$C_{15}H_{20}O_{2}N$	0.78	4.85	pHLM and rat
		FI at *m/z* 189.0912	189.0910	$C_{12}H_{13}O_{2}$	0.95		
		FI at *m/z* 161.0962	161.0961	$C_{11}H_{13}O$	0.43		
		FI at *m/z* 107.0496	107.0491	$C_{7}H_{7}O$	3.98		
		FI at *m/z* 58.0660	58.6051	$C_{3}H_{8}N$	15.2		
CM5	*N*-Dealkylation + glucuronidation	PI at *m/z* 366.1552	366.1547	$C_{18}H_{24}O_{7}N$	1.15	5.35	rat
		FI at *m/z* 348.1449	348.1442	$C_{18}H_{22}O_{6}N$	-1.73		
		FI at *m/z* 330.1336	330.1336	$C_{18}H_{20}O_{5}N$	-0.77		
		FI at *m/z* 173.0964	173.0961	$C_{12}H_{13}O$	1.72		
		FI at *m/z* 145.1014	145.1012	$C_{11}H_{13}$	1.29		
		FI at *m/z* 129.0701	129.0699	$C_{10}H_{9}$	1.60		
		FI at *m/z* 91.0548	91.0542	$C_{7}H_{7}$	6.73		
		FI at *m/z* 67.0551	67.0542	$C_{5}H_{7}$	13.4		

Table S2. List of *in vivo* phase I and II metabolites of 2-oxo-PCE, identified in rat urine samples and *in vitro* phase I metabolites identified in incubations using pooled human liver microsomes (pHLM), including the respective metabolite ID, metabolic reaction, masses of the precursor ion (PI) and characteristic fragment ions (FI) detected in MS^2, calculated exact masses, elemental composition, calculated mass errors in parts per million (ppm), retention times (RT) in minutes, and system in which metabolites were identified. The metabolites are sorted by their mass and RT. pHLM, identified in pHLM incubations; rat, identified in rat urine samples

Metabolite ID	Metabolic Reaction	Characteristic Ions Measured Accurate Masses	Calculated Exact Masses, *m/z*	Elemental Composition	Error, ppm	RT, min	Identified in
2-Oxo-PCE	Parent compound	PI at *m/z* 218.1540	218.1539	$C_{14}H_{20}ON$	-2.21	5.44	pHLM and rat
		FI at *m/z* 200.1429	200.1434	$C_{14}H_{18}N$	-2.54		
		FI at *m/z* 173.0957	173.0961	$C_{12}H_{13}O$	-1.98		
		FI at *m/z* 155.0851	155.0855	$C_{12}H_{11}$	-3.06		
		FI at *m/z* 145.1009	145.1012	$C_{11}H_{13}$	-2.18		
		FI at *m/z* 129.0697	129.0699	$C_{10}H_{9}$	-1.12		
		FI at *m/z* 117.0699	117.0699	$C_{9}H_{9}$	-0.12		
		FI at *m/z* 91.0545	91.0542	$C_{7}H_{7}$	3.38		
		FI at *m/z* 67.0549	67.0542	$C_{5}H_{7}$	9.37		
EM1	*N*-Dealkylation	PI at *m/z* 190.1225	190.1226	$C_{12}H_{16}ON$	-0.78	5.04	pHLM and rat
		FI at *m/z* 173.0961	173.0961	$C_{12}H_{13}O$	0.14		
		FI at *m/z* 155.0854	155.0855	$C_{12}H_{11}$	-0.60		
		FI at *m/z* 145.1012	145.1012	$C_{11}H_{13}$	0.03		
		FI at *m/z* 129.0700	129.0699	$C_{10}H_{9}$	0.89		
		FI at *m/z* 117.0701	117.0699	$C_{9}H_{9}$	2.29		
		FI at *m/z* 91.0547	91.0542	$C_{7}H_{7}$	5.65		
		FI at *m/z* 67.0550	67.0542	$C_{5}H_{7}$	11.6		
EM2	Hydroxylation + oxidation to a ketone	PI at *m/z* 232.1333	232.1332	$C_{14}H_{18}O_{2}N$	0.61	4.75	rat
		FI at *m/z* 187.0754	187.0754	$C_{12}H_{11}O_{2}$	0.28		
		FI at *m/z* 159.0805	159.0804	$C_{11}H_{11}O$	0.11		
		FI at *m/z* 91.0547	91.0542	$C_{7}H_{7}$	5.48		
EM3	Hydroxylation isomer 1	PI at *m/z* 234.1491	234.1489	$C_{14}H_{20}O_{2}N$	1.21	4.11	pHLM and rat
		FI at *m/z* 216.1389	216.1383	$C_{14}H_{18}ON$	2.96		
		FI at *m/z* 189.0910	189.0910	$C_{12}H_{13}O_{2}$	-0.01		
		FI at *m/z* 171.0805	171.0804	$C_{12}H_{11}O$	0.37		
		FI at *m/z* 161.0963	161.0961	$C_{11}H_{13}O$	1.57		
		FI at *m/z* 143.0856	143.0855	$C_{11}H_{11}$	0.84		
		FI at *m/z* 129.0701	129.0699	$C_{10}H_{9}$	1.84		
		FI at *m/z* 91.0548	91.0542	$C_{7}H_{7}$	6.23		
EM4	Hydroxylation isomer 2	PI at *m/z* 234.1490	234.1489	$C_{14}H_{20}O_{2}N$	0.63	4.62	pHLM and rat
		FI at *m/z* 216.1381	216.1383	$C_{14}H_{18}ON$	-0.84		
		FI at *m/z* 189.0912	189.0910	$C_{12}H_{13}O_{2}$	1..12		
		FI at *m/z* 171.0805	171.0804	$C_{12}H_{11}O$	0.28		
		FI at *m/z* 161.0961	161.0961	$C_{11}H_{13}O$	-0.23		
		FI at *m/z* 143.0856	143.0855	$C_{11}H_{11}$	0.84		
		FI at *m/z* 129.0701	129.0699	$C_{10}H_{9}$	1.47		
		FI at *m/z* 91.0548	91.0542	$C_{7}H_{7}$	6.57		
EM5	*N*-Dealkylation + glucuronidation	PI at *m/z* 366.1546	366.1547	$C_{18}H_{24}O_{7}N$	-0.26	5.20	rat
		FI at *m/z* 348.1444	348.1442	$C_{18}H_{22}O_{6}N$	0.61		
		FI at *m/z* 330.1329	330.1336	$C_{18}H_{20}O_{5}N$	-2.01		
		FI at *m/z* 173.0963	173.0961	$C_{12}H_{13}O$	0.93		
		FI at *m/z* 145.1012	145.1012	$C_{11}H_{13}$	0.45		
		FI at *m/z* 129.0703	129.0699	$C_{10}H_{9}$	3.14		
		FI at *m/z* 91.0548	91.0542	$C_{7}H_{7}$	6.32		
		FI at *m/z* 67.0550	67.0542	$C_{5}H_{7}$	11.9		

Table S3. List of *in vivo* phase I and II metabolites of 2-oxo-PCiP, identified in rat urine samples and *in vitro* phase I metabolites identified in incubations using pooled human liver microsomes (pHLM), including the respective metabolite ID, metabolic reaction, masses of the precursor ion (PI) and characteristic fragment ions (FI) detected in MS^2, calculated exact masses, elemental composition, calculated mass errors in parts per million (ppm), retention times (RT) in minutes, and system in which metabolites were identified. The metabolites are sorted by their mass and RT. pHLM, identified in pHLM incubations; rat, identified in rat urine samples

Metabolite ID	Metabolic Reaction	Characteristic Ions Measured Accurate Masses	Calculated Exact Masses, *m/z*	Elemental Composition	Error, ppm	RT, min	Identified in
2-Oxo-PCiP	Parent compound	PI at *m/z* 232.1690	232.1696	$C_{15}H_{22}ON$	-2.71	5.95	pHLM and rat
		FI at m/z 214.1596	214.1590	$C_{15}H_{20}N$	2.50		
		FI at *m/z* 173.0958	173.0961	$C_{12}H_{13}O$	-1.54		
		FI at *m/z* 155.0852	155.0855	$C_{12}H_{11}$	-2.17		
		FI at *m/z* 145.1009	145.1012	$C_{11}H_{13}$	-2.08		
		FI at *m/z* 129.0697	129.0699	$C_{10}H_{9}$	-1.00		
		FI at *m/z* 117.0700	117.0699	$C_{9}H_{9}$	0.66		
		FI at *m/z* 91.0546	91.0542	$C_{7}H_{7}$	3.72		
		FI at *m/z* 60.0815	60.0808	$C_{3}H_{10}N$	12.1		
IM1	*N*-Dealkylation	PI at *m/z* 190.1229	190.1226	$C_{12}H_{16}ON$	1.49	5.03	pHLM and rat
		FI at *m/z* 173.0962	173.0961	$C_{12}H_{13}O$	0.66		
		FI at *m/z* 155.0856	155.0855	$C_{12}H_{11}$	0.29		
		FI at *m/z* 145.1012	145.1012	$C_{11}H_{13}$	0.45		
		FI at *m/z* 129.0701	129.0699	$C_{10}H_{9}$	1.48		
		FI at *m/z* 117.0702	117.0699	$C_{9}H_{9}$	2.42		
		FI at *m/z* 91.0548	91.0542	$C_{7}H_{7}$	6.07		
		FI at *m/z* 67.0550	67.0542	$C_{5}H_{7}$	12.1		
IM2	*N*-Dealkylation + hydroxylation	PI at *m/z* 206.1175	206.1176	$C_{12}H_{16}O_{2}N$	-0.38	4.78	pHLM and rat
		FI at *m/z* 189.0910	189.0910	$C_{12}H_{13}O_{2}$	-0.01		
		FI at *m/z* 171.0804	171.0804	$C_{12}H_{11}O$	0.02		
		FI at *m/z* 161.0961	161.0961	$C_{11}H_{13}O$	-0.04		
		FI at *m/z* 143.0856	143.0855	$C_{11}H_{11}$	0.31		
		FI at *m/z* 129.0701	129.0699	$C_{10}H_{9}$	1.37		
		FI at *m/z* 91.0547	91.0542	$C_{7}H_{7}$	5.64		
IM3	Hydroxylation isomer 1 + oxidation to a ketone	PI at *m/z* 246.1490	246.1489	$C_{15}H_{20}O_{2}N$	0.66	5.22	rat
		FI at *m/z* 187.0755	187.0754	$C_{12}H_{11}O_{2}$	0.77		
		FI at *m/z* 159.0805	159.0804	$C_{11}H_{11}O$	0.40		
		FI at *m/z* 91.0547	91.0542	$C_{7}H_{7}$	5.48		
		FI at *m/z* 60.0817	60.0808	$C_{3}H_{10}N$	14.6		
IM4	Hydroxylation isomer 1	PI at *m/z* 248.1644	248.1645	$C_{12}H_{22}O_{2}N$	-0.61	4.75	pHLM and rat
		FI at *m/z* 230.1541	230.1539	$C_{12}H_{20}ON$	0.81		
		FI at *m/z* 189.0912	189.0910	$C_{12}H_{13}O_{2}$	0.87		
		FI at *m/z* 171.0805	171.0804	$C_{12}H_{11}O$	0.28		
		FI at *m/z* 143.0856	143.0855	$C_{11}H_{11}$	0.84		
		FI at *m/z* 91.0548	91.0542	C7H7	6.15		
		FI at *m/z* 60.0817	60.0808	$C_{3}H_{10}N$	15.1		
IM5	Hydroxylation isomer 2	PI at *m/z* 248.1645	248.1645	$C_{15}H_{22}O_{2}N$	0.23	5.04	pHLM and rat
		FI at *m/z* 189.0910	189.0910	$C_{12}H_{13}O$	-0.18		
		FI at *m/z* 161.0961	161.0961	$C_{11}H_{13}O$	-0.09		
		FI at *m/z* 107.0496	107.0491	$C_{7}H_{7}O$	4.30		
		FI at *m/z* 60.0816	60.0808	$C_{3}H_{10}N$	14.2		

IM6	Hydroxylation	PI at m/z 248.1646	248.1645	$C_{15}H_{22}O_2N$	0.55	5.58	pHLM and rat
	isomer 3	FI at m/z 230.1541	230.1539	$C_{15}H_{20}ON$	0.69		
		FI at m/z 173.0963	173.0961	$C_{12}H_{13}O$	1.02		
		FI at m/z 145.1013	145.1012	$C_{11}H_{13}$	0.66		
		FI at m/z 129.0700	129.0699	$C_{10}H_9$	1.13		
		FI at m/z 91.0548	91.0542	C_7H_7	6.23		
		FI at m/z 76.0764	76.0757	$C_3H_{10}ON$	9.24		
		FI at m/z 58.0660	58.0651	C_3H_8N	15.4		
IM7	Dihydroxylation	PI at m/z 264.1593	264.1645	$C_{15}H_{22}O_3N$	-0.45	4.61	rat
		FI at m/z 246.1490	246.1489	$C_{15}H_{20}O_2N$	0.66		
		FI at m/z 228.1383	228.1383	$C_{15}H_{18}ON$	-0.07		
		FI at m/z 189.0908	189.0910	$C_{12}H_{13}O_2$	-1.14		
		FI at m/z 171.0805	171.0804	$C_{12}H_{11}O$	0.37		
		FI at m/z 143.0856	143.0855	$C_{11}H_{11}$	0.63		
		FI at m/z 76.0764	76.0757	$C_3H_{10}ON$	9.94		
		FI at m/z 58.0660	58.0651	C_3H_8N	15.4		
IM8	*N*-Dealkylation +	PI at m/z 366.1543	366.1547	$C_{18}H_{24}O_7N$	-1.18	5.22	rat
	glucuronidation	FI at m/z 348.1441	348.1442	$C_{18}H_{22}O_6N$	-0.08		
		FI at m/z 330.1340	330.1336	$C_{18}H_{20}O_5N$	1.17		
		FI at m/z 173.0962	173.0961	$C_{12}H_{13}O$	0.47		
		FI at m/z 145.1012	145.1012	$C_{11}H_{13}$	0.14		
		FI at m/z 129.0701	129.0699	$C_{10}H_9$	1.58		
		FI at m/z 91.0548	91.0542	C_7H_7	6.58		
IM9	Hydroxylation	PI at m/z 424.1966	424.1966	$C_{21}H_{30}O_8N$	0.00	5.24	rat
	isomer 3 +	FI at m/z 230.1537	230.1539	$C_{15}H_{20}ON$	-0.85		
	glucuronidation	FI at m/z 173.0963	173.0961	$C_{12}H_{13}O$	1.28		
		FI at m/z 145.1013	145.1012	$C_{12}H_{13}O$	0.72		
		FI at m/z 129.0703	129.0699	$C_{10}H_9$	3.06		
		FI at m/z 91.0548	91.0542	C_7H_7	6.62		
		FI at m/z 76.0764	76.0757	$C_3H_{10}ON$	8.90		

Table S4. List of *in vivo* phase I and II metabolites of 2-oxo-PCMe, identified in rat urine samples and *in vitro* phase I metabolites identified in incubations using pooled human liver microsomes (pHLM), including the respective metabolite ID, metabolic reaction, masses of the precursor ion (PI) and characteristic fragment ions (FI) detected in MS², calculated exact masses, elemental composition, calculated mass errors in parts per million (ppm), retention times (RT) in minutes, and system in which metabolites were identified. The metabolites are sorted by their mass and RT. pHLM, identified in pHLM incubations; rat, identified in rat urine samples

Metabolite ID	Metabolic Reaction	Characteristic Ions Measured Accurate Masses	Calculated Exact Masses, *m/z*	Elemental Composition	Error, ppm	RT, min	Identified in
2-Oxo-PCMe	Parent	PI at m/z 204.1383	204.1383	$C_{13}H_{18}ON$	0.15	5.10	pHLM and rat
	compound	FI at m/z 186.1277	186.1277	$C_{13}H_{16}N$	0.03		
		FI at m/z 173.0962	173.0961	$C_{12}H_{13}O$	0.75		
		FI at m/z 155.0855	155.0855	$C_{12}H_{11}$	-0.01		
		FI at m/z 145.1012	145.1012	$C_{11}H_{13}$	0.45		
		FI at m/z 129.0700	129.0699	$C_{10}H_9$	1.13		
		FI at m/z 117.0702	117.0699	C_9H_9	2.87		
		FI at m/z 91.0548	91.0542	C_7H_7	6.07		
		FI at m/z 67.0551	67.0542	C_5H_7	12.3		
MM1	*N*-Dealkylation	PI at m/z 190.1222	190.1226	$C_{12}H_{16}ON$	-2.28	5.00	pHLM and rat
		FI at m/z 173.0962	173.0961	$C_{12}H_{13}O$	0.84		
		FI at m/z 155.0856	155.0855	$C_{12}H_{11}$	0.48		
		FI at m/z 145.1013	145.1012	$C_{11}H_{13}$	0.69		
		FI at m/z 129.0701	129.0699	$C_{10}H_9$	1.72		
		FI at m/z 117.0703	117.0699	C_9H_9	3.33		
		FI at m/z 91.0548	91.0542	C_7H_7	6.23		
		FI at m/z 67.0551	67.0542	C_5H_7	12.5		
MM2	*N*-Dealkylation +	PI at m/z 206.1181	206.1176	$C_{12}H_{16}O_2N$	2.73	4.82	pHLM and rat
	hydroxylation	FI at m/z 189.0912	189.0910	$C_{12}H_{13}O_2$	0.87		
		FI at m/z 171.0805	171.0804	$C_{12}H_{11}O$	0.55		
		FI at m/z 161.0962	161.0961	$C_{11}H_{13}O$	0.71		
		FI at m/z 143.0857	143.0855	$C_{11}H_{11}$	0.95		
		FI at m/z 129.0701	129.0699	$C_{10}H_9$	1.48		
		FI at m/z 117.0702	117.0699	C_9H_9	2.87		
		FI at m/z 91.0548	91.0542	C_7H_7	6.32		
		FI at m/z 67.0548	67.0542	C_5H_7	8.69		
MM3	*N*-Dealkylation +	PI at m/z 206.1177	206.1176	$C_{12}H_{16}O_2N$	0.88	5.61	rat
	hydroxylamine	FI at m/z 188.1071	188.1070	$C_{12}H_{14}ON$	0.51		
		FI at m/z 173.0962	173.0961	$C_{12}H_{13}O$	0.58		
		FI at m/z 145.1012	145.1012	$C_{11}H_{13}$	0.13		
		FI at m/z 129.0701	129.0699	$C_{10}H_9$	1.37		
		FI at m/z 117.0702	117.0699	C_9H_9	2.35		
		FI at m/z 91.0548	91.0542	C_7H_7	5.81		
		FI at m/z 67.0550	67.0542	C_5H_7	12.0		
MM4	Hydroxylation	PI at m/z 218.1172	218.1176	$C_{13}H_{16}O_2N$	-1.48	4.61	rat
	isomer 1 +	FI at m/z 187.0751	187.0754	$C_{12}H_{11}O_2$	-1.27		
	oxidation to a	FI at m/z 159.0802	159.0804	$C_{11}H_{11}O$	-1.23		
	ketone	FI at m/z 91.0546	91.0542	C_7H_7	4.56		
MM5	Hydroxylation	PI at m/z 220.1330	220.1332	$C_{13}H_{18}O_2N$	-0.88	4.49	pHLM and rat
	isomer 1	FI at m/z 202.1223	202.1226	$C_{13}H_{16}ON$	-1.61		
		FI at m/z 189.0910	189.0910	$C_{12}H_{13}O_2$	-0.01		
		FI at m/z 171.0804	171.0804	$C_{12}H_{11}O$	-0.07		
		FI at m/z 143.0856	143.0855	$C_{11}H_{11}$	0.52		
		FI at m/z 91.0548	91.0542	C_7H_7	5.98		

MM6	Hydroxylation isomer 2	PI at m/z 220.1335	220.1332	$C_{13}H_{18}O_2N$	1.27	4.70	pHLM and rat
		FI at m/z 189.0911	189.0910	$C_{12}H_{13}O_2$	0.55		
		FI at m/z 161.0961	161.0961	$C_{11}H_{13}O$	0.34		
		FI at m/z 107.0496	107.0491	C_7H_7O	4.13		
		FI at m/z 67.0551	67.0542	C_5H_7	12.4		
MM7	*N*-Dealkylation + acetylation	PI at m/z 232.1341	232.1332	$C_{14}H_{18}O_2N$	3.83	6.90	rat
		FI at m/z 190.1231	190.1226	$C_{12}H_{16}ON$	2.62		
		FI at m/z 173.0962	173.0961	$C_{12}H_{13}O$	0.75		
		FI at m/z 155.0853	155.0855	$C_{12}H_{11}$	-1.58		
		FI at m/z 145.1013	145.1012	$C_{11}H_{13}$	0.66		
		FI at m/z 129.0697	129.0699	$C_{10}H_9$	-1.00		
		FI at m/z 91.0548	91.0542	C_7H_7	6.23		
		FI at m/z 67.0550	67.0542	C_5H_7	12.0		
MM8	*N*-Dealkylation + glucuronidation	PI at m/z 366.1552	366.1547	$C_{18}H_{24}O_7N$	1.15	5.25	rat
		FI at m/z 348.1444	348.1442	$C_{18}H_{22}O_6N$	0.70		
		FI at m/z 330.1332	330.1336	$C_{18}H_{20}O_5N$	-1.28		
		FI at m/z 173.0963	173.0961	$C_{12}H_{13}O$	1.11		
		FI at m/z 155.0856	155.0855	$C_{12}H_{11}$	0.19		
		FI at m/z 145.1013	145.1012	$C_{11}H_{13}$	0.76		
		FI at m/z 129.0701	129.0699	$C_{10}H_9$	1.37		
		FI at m/z 91.0548	91.0542	C_7H_7	6.57		
		FI at m/z 67.0551	67.0542	C_5H_7	12.9		
MM9	*N*-Dealkylation + hydroxylation glucuronidation	PI at m/z 382.1491	382.1469	$C_{18}H_{24}O_8N$	-1.52	4.37	rat
		FI at m/z 364.1381	364.1391	$C_{18}H_{22}O_7N$	-2.59		
		FI at m/z 171.0803	171.0804	$C_{12}H_{11}O$	-0.91		
		FI at m/z 161.0960	161.0961	$C_{11}H_{13}O$	-0.79		
		FI at m/z 143.0856	143.0855	$C_{11}H_{11}$	0.29		
		FI at m/z 91.0553	91.0542	C_7H_7	11.5		

Table S5. List of *in vivo* phase I and II metabolites of 2-oxo-PCPr, identified in rat urine samples and *in vitro* phase I metabolites identified in incubations using pooled human liver microsomes (pHLM), including the respective metabolite ID, metabolic reaction, masses of the precursor ion (PI) and characteristic fragment ions (FI) detected in MS², calculated exact masses, elemental composition, calculated mass errors in parts per million (ppm), retention times (RT) in minutes, and system in which metabolites were identified. The metabolites are sorted by their mass and RT. pHLM, identified in pHLM incubations; rat, identified in rat urine samples

Metabolite ID	Metabolic Reaction	Characteristic Ions Measured Accurate Masses	Calculated Exact Masses, *m/z*	Elemental Composition	Error, ppm	RT, min	Identified in
2-Oxo-PCPr	Parent compound	PI at m/z 232.1690	232.1696	$C_{15}H_{22}ON$	-2.39	5.96	pHLM and rat
		FI at m/z 214.1587	214.1590	$C_{15}H_{20}N$	-1.71		
		FI at m/z 173.0958	173.0961	$C_{12}H_{13}O$	-1.54		
		FI at m/z 155.0851	155.0855	$C_{12}H_{11}$	-2.47		
		FI at m/z 145.1009	145.1012	$C_{11}H_{13}$	-1.87		
		FI at m/z 129.0697	129.0699	$C_{10}H_{9}$	-1.71		
		FI at m/z 117.0700	117.0699	$C_{9}H_{9}$	1.31		
		FI at m/z 91.0546	91.0542	$C_{7}H_{7}$	3.89		
		FI at m/z 60.0815	60.0808	$C_{3}H_{10}N$	12.4		
PM1	*N*-Dealkylation	PI at m/z 190.1223	190.1226	$C_{12}H_{16}ON$	-1.82	5.05	pHLM and rat
		FI at m/z 173.0962	173.0961	$C_{12}H_{13}O$	0.63		
		FI at m/z 155.0854	155.0855	$C_{12}H_{11}$	-1.05		
		FI at m/z 145.1012	145.1012	$C_{11}H_{13}$	0.17		
		FI at m/z 129.0700	129.0699	$C_{10}H_{9}$	0.80		
		FI at m/z 117.0702	117.0699	$C_{9}H_{9}$	2.35		
		FI at m/z 91.0548	91.0542	$C_{7}H_{7}$	5.99		
		FI at m/z 67.0550	67.0542	$C_{5}H_{7}$	11.5		
PM2	Hydroxylation isomer 1 + oxidation to a ketone	PI at m/z 246.1490	246.1489	$C_{15}H_{20}O_{2}N$	0.66	5.37	rat
		FI at m/z 228.1380	228.1383	$C_{15}H_{18}ON$	-1.07		
		FI at m/z 187.0753	187.0754	$C_{12}H_{11}O_{2}$	-0.53		
		FI at m/z 159.0804	159.0804	$C_{11}H_{11}O$	-0.46		
		FI at m/z 141.0698	141.0699	$C_{11}H_{9}$	-0.48		
		FI at m/z 91.0546	91.0542	$C_{7}H_{7}$	4.14		
		FI at m/z 60.0816	60.0808	$C_{3}H_{10}N$	14.2		
PM3	Hydroxylation isomer 1	PI at m/z 248.1639	248.1645	$C_{15}H_{22}O_{2}N$	-2.52	4.75	pHLM and rat
		FI at m/z 230.1540	230.1539	$C_{15}H_{20}ON$	0.15		
		FI at m/z 189.0912	189.0910	$C_{12}H_{13}O_{2}$	0.87		
		FI at m/z 171.0805	171.0804	$C_{12}H_{11}O$	0.28		
		FI at m/z 143.0857	143.0855	$C_{11}H_{11}$	0.95		
		FI at m/z 60.0817	60.0808	$C_{3}H_{10}N$	17.8		
PM4	Hydroxylation isomer 2	PI at m/z 248.1649	248.1645	$C_{15}H_{22}O_{2}N$	1.41	5.31	pHLM and rat
		FI at m/z 230.1537	230.1539	$C_{15}H_{20}ON$	-0.91		
		FI at m/z 173.0963	173.0961	$C_{12}H_{13}O$	1.02		
		FI at m/z 155.0854	155.0855	$C_{12}H_{11}$	-0.50		
		FI at m/z 145.1012	145.1012	$C_{11}H_{13}$	0.34		
		FI at m/z 129.0701	129.0699	$C_{10}H_{9}$	1.96		
		FI at m/z 117.0702	117.0699	$C_{9}H_{9}$	2.94		
		FI at m/z 91.0548	91.0542	$C_{7}H_{7}$	6.07		
		FI at m/z 76.0764	76.0757	$C_{3}H_{10}ON$	9.44		
		FI at m/z 67.0550	67.0542	$C_{5}H_{7}$	12.2		

PM5	Hydroxylamine	PI at m/z 248.1648	248.1645	$C_{15}H_{22}O_2N$	1.29	6.21	pHLM and rat
		FI at m/z 230.1541	230.1539	$C_{15}H_{20}ON$	0.55		
		FI at m/z 173.0962	173.0961	$C_{12}H_{13}O$	0.40		
		FI at m/z 155.0860	155.0855	$C_{12}H_{11}$	3.04		
		FI at m/z 145.1013	145.1012	$C_{11}H_{13}$	0.69		
		FI at m/z 129.0701	129.0699	$C_{10}H_9$	1.72		
		FI at m/z 91.0548	91.0542	C_7H_7	6.65		
		FI at m/z 67.0550	67.0542	C_5H_7	11.3		
PM6	Dihydroxylation isomer 1 + monooxoidation to a ketone	PI at m/z 262.1438	262.1438	$C_{15}H_{20}O_3N$	0.11	4.85	rat
		FI at m/z 244.1321	244.1332	$C_{15}H_{18}O_2N$	-4.61		
		FI at m/z 187.0754	187.0754	$C_{12}H_{11}O_2$	0.28		
		FI at m/z 159.0805	159.0804	$C_{11}H_{11}O$	0.21		
		FI at m/z 76.0764	76.0757	$C_3H_{10}ON$	9.64		
PM7	Hydroxylation isomer 2 + oxidation to carboxylic acid	PI at m/z 262.1440	262.1438	$C_{15}H_{20}O_3N$	0.69	5.31	rat
		FI at m/z 244.1338	244.1332	$C_{15}H_{18}O_2N$	2.33		
		FI at m/z 173.0962	173.0961	$C_{12}H_{13}O$	0.58		
		FI at m/z 155.0855	155.0855	$C_{12}H_{11}$	-0.11		
		FI at m/z 145.1012	145.1012	$C_{11}H_{13}$	0.45		
		FI at m/z 129.0700	129.0699	$C_{10}H_9$	0.77		
		FI at m/z 117.0702	117.0699	C_9H_9	2.87		
		FI at m/z 91.0548	91.0542	C_7H_7	5.98		
		FI at m/z 67.0660	67.0542	C_5H_7	12.2		
PM8	Dihydroxylation isomer 1	PI at m/z 264.1596	264.1594	$C_{15}H_{22}O_3N$	0.82	4.61	rat
		FI at m/z 246.1490	246.1489	$C_{15}H_{20}O_2N$	0.72		
		FI at m/z 228.1382	228.1383	$C_{15}H_{18}ON$	-0.60		
		FI at m/z 189.0911	189.0910	$C_{12}H_{13}O_2$	0.31		
		FI at m/z 171.0805	171.0804	$C_{12}H_{11}O$	0.55		
		FI at m/z 143.0857	143.0855	$C_{11}H_{11}$	1.06		
		FI at m/z 76.0764	76.0757	$C_3H_{10}ON$	9.94		
PM9	Dihydroxylation isomer 2	PI at m/z 264.1595	264.1594	$C_{15}H_{22}O_3N$	0.36	5.09	rat
		FI at m/z 246.1490	246.1489	$C_{15}H_{20}O_2N$	0.72		
		FI at m/z 189.0910	189.0910	$C_{12}H_{13}O_2$	-0.01		
		FI at m/z 161.0961	161.0961	$C_{11}H_{13}O$	0.15		
		FI at m/z 107.0495	107.0491	C_7H_7O	3.77		
		FI at m/z 76.0764	76.0757	$C_3H_{10}ON$	9.45		
PM10	*N*-Dealkylation + glucuronidation	PI at m/z 366.1547	366.1547	$C_{18}H_{24}O_7N$	-0.01	5.29	rat
		FI at m/z 348.1437	348.1442	$C_{18}H_{22}O_6N$	-1.22		
		FI at m/z 330.1341	330.1336	$C_{18}H_{20}O_5N$	1.50		
		FI at m/z 173.0962	173.0961	$C_{12}H_{13}O$	0.66		
		FI at m/z 145.1013	145.1012	$C_{11}H_{13}$	0.66		
		FI at m/z 129.0701	129.0699	$C_{10}H_9$	1.72		
		FI at m/z 91.0548	91.0542	C_7H_7	6.23		
PM11	Hydroxylation isomer 2 + glucuronidation	PI at m/z 424.1964	424.1966	$C_{21}H_{30}O_8N$	-0.36	5.24	rat
		FI at m/z 248.1640	248.1645	$C_{15}H_{22}O_2N$	-1.85		
		FI at m/z 230.1539	230.1539	$C_{15}H_{20}ON$	-0.31		
		FI at m/z 173.0965	173.0961	$C_{12}H_{13}O$	2.52		
		FI at m/z 145.1012	145.1012	$C_{11}H_{13}$	0.34		
		FI at m/z 129.0702	129.0699	$C_{10}H_9$	2.19		
		FI at m/z 91.0548	91.0542	C_7H_7	6.23		
		FI at m/z 76.0764	76.0757	$C_3H_{10}ON$	9.73		

Table S6. Parent compounds and metabolites of five deschloroketamine derivatives detected by GC-MS, including masses of precursor ions (PI), elemental composition, and masses of characteristic fragment ions (FI). AC, acetylated.

Parent compound or metabolite	PI mass, *m/z*	RI	Elemental composition	Characteristic FI
2-Oxo-PCcP	229	1760	$C_{15}H_{19}NO$	200, 172, 145, 104, 91
2-Oxo-PCE	217	1635	$C_{14}H_{19}NO$	189, 160, 146, 132, 117, 104, 91
2-Oxo-PCiP	231	1648	$C_{15}H_{21}NO$	203, 174, 160, 132, 117, 104, 91
2-Oxo-PCMe AC	245	1990	$C_{15}H_{19}NO_2$	217, 174, 160, 144, 132, 118, 104, 91
2-Oxo-PCPr	231	1747	$C_{15}H_{21}NO$	203, 174, 160, 132, 117, 104, 91
2-Oxo-PCPr AC	273	2038	$C_{15}H_{21}NO$	245, 203, 174, 160, 144, 132, 117, 104, 91
N-Dealkylation AC	231	1874	$C_{14}H_{17}NO_2$	188, 174, 144, 132, 104, 91

Table S7. 2-Oxo-PCcP and its metabolites detected in rat urine after oral administration using different sample preparations in combination with LC-HRMS/MS (#) or GC-MS (*). Metabolite IDs correspond to Tables S1. CM, 2-oxo-PCcP metabolite; UPP, urine precipitation; UGLUC, urine after glucuronidase/arylsulfatase cleavage; LLE, liquid-liquid extraction; SPE, solid-phase extraction; UHyAc, partial urine hydrolysis followed by LLE and acetylation

Metabolite ID	Sample preparation performed				
	**UPP#**	**UGLUC LLE#**	**SPE#**	**UGLUC SPE#**	**UHyAC***
2-oxo-PCcP	not detected	not detected	detected	detected	not detected
CM1	detected	detected	detected	detected	detected
CM2	not detected	not detected	detected	not detected	not detected
CM3	not detected	detected	detected	detected	not detected
CM4	not detected	detected	detected	detected	not detected
CM5	detected	not detected	detected	not detected	not detected
Summary	2 of 6	3 of 6	6 of 6	4 of 6	1 of 6

Table S8. 2-Oxo-PCE and its metabolites detected in rat urine after oral administration using different sample preparations in combination with LC-HRMS/MS (#) or GC-MS (*). Metabolite IDs correspond to Table S2. EM, 2-oxo-PCE metabolite; UPP, urine precipitation; UGLUC, urine after glucuronidase/arylsulfatase cleavage; LLE, liquid-liquid extraction; SPE, solid-phase extraction; UHyAc, partial urine hydrolysis followed by LLE and acetylation

Metabolite ID	Sample preparation performed				
	**UPP#**	**UGLUC LLE#**	**SPE#**	**UGLUC SPE#**	**UHyAC***
2-Oxo-PCE	detected	detected	detected	detected	not detected
EM1	detected	detected	detected	detected	detected
EM2	detected	detected	detected	detected	not detected
EM3	detected	detected	detected	detected	not detected
EM4	detected	detected	detected	detected	not detected
EM5	detected	not detected	detected	not detected	not detected
Summary	6 of 6	5 of 6	6 of 6	5 of 6	1 of 6

Table S9. 2-Oxo-PCiP and its metabolites detected in rat urine after oral administration using different sample preparations in combination with LC-HRMS/MS (#) or GC-MS (*). Metabolite IDs correspond to Table S3. IM, 2-oxo-PCiP metabolite; UPP, urine precipitation; UGLUC, urine after glucuronidase/arylsulfatase cleavage; LLE, liquid-liquid extraction; SPE, solid-phase extraction; UHyAc, partial urine hydrolysis followed by LLE and acetylation

Metabolite ID	Sample preparation performed				
	**UPP[#]**	**UGLUC LLE[#]**	**SPE[#]**	**UGLUC SPE[#]**	**UHyAC[*]**
2-Oxo-PCiP	not detected	detected	detected	detected	not detected
IM1	detected	detected	detected	detected	detected
IM2	not detected	detected	detected	detected	not detected
IM3	not detected	not detected	detected	not detected	not detected
IM4	detected	detected	detected	detected	not detected
IM5	not detected	detected	not detected	detected	not detected
IM6	not detected	detected	not detected	detected	not detected
IM7	not detected	not detected	detected	not detected	not detected
IM8	detected	not detected	detected	not detected	not detected
IM9	detected	not detected	detected	not detected	not detected
Summary	4 of 10	6 of 10	8 of 10	6 of 10	1 of 10

Table S10. 2-Oxo-PCMe and its metabolites detected in rat urine after oral administration using different sample preparations in combination with LC-HRMS/MS (#) or GC-MS (*). Metabolite IDs correspond to Table S4. MM, 2-oxo-PCMe metabolite; UPP, urine precipitation; UGLUC, urine after glucuronidase/arylsulfatase cleavage; LLE, liquid-liquid extraction; SPE, solid-phase extraction; UHyAc, partial urine hydrolysis followed by LLE and acetylation

Metabolite ID	Sample preparation performed				
	**UPP[#]**	**UGLUC LLE[#]**	**SPE[#]**	**UGLUC SPE[#]**	**UHyAC[*]**
2-Oxo-PCMe	detected	detected	detected	detected	not detected
MM1	detected	detected	detected	detected	detected
MM2	detected	detected	detected	detected	not detected
MM3	detected	detected	detected	detected	not detected
MM4	detected	detected	detected	detected	not detected
MM5	detected	detected	detected	detected	not detected
MM6	not detected	detected	detected	detected	not detected
MM7	not detected	not detected	detected	not detected	not detected
MM8	detected	not detected	detected	not detected	not detected
MM9	not detected	not detected	detected	not detected	not detected
Summary	7 of 10	7 of 10	10 of 10	7 of 10	1 of 10

Table S11. 2-Oxo-PCPr and its metabolites detected in rat urine after oral administration using different sample preparations in combination with LC-HRMS/MS (#) or GC-MS (*). Metabolite IDs correspond to Table S5. PM, 2-oxo-PCPr metabolite; UPP, urine precipitation; UGLUC, urine after glucuronidase/arylsulfatase cleavage; LLE, liquid-liquid extraction; SPE, solid-phase extraction; UHyAc, partial urine hydrolysis followed by LLE and acetylation

Metabolite ID	Sample preparation performed				
	UPP#	UGLUC LLE#	SPE#	UGLUC SPE#	UHyAC*
2-Oxo-PCPr	detected	detected	detected	detected	not detected
PM1	detected	detected	detected	detected	detected
PM2	detected	detected	detected	detected	not detected
PM3	detected	detected	detected	detected	not detected
PM4	detected	detected	detected	detected	not detected
PM5	detected	detected	detected	detected	not detected
PM6	detected	detected	detected	detected	not detected
PM7	detected	detected	detected	detected	not detected
PM8	not detected	detected	detected	detected	not detected
PM9	detected	detected	detected	detected	not detected
PM10	detected	not detected	detected	not detected	not detected
PM11	not detected	not detected	detected	not detected	not detected
Summary	10 of 12	10 of 12	12 of 12	10 of 12	1 of 12

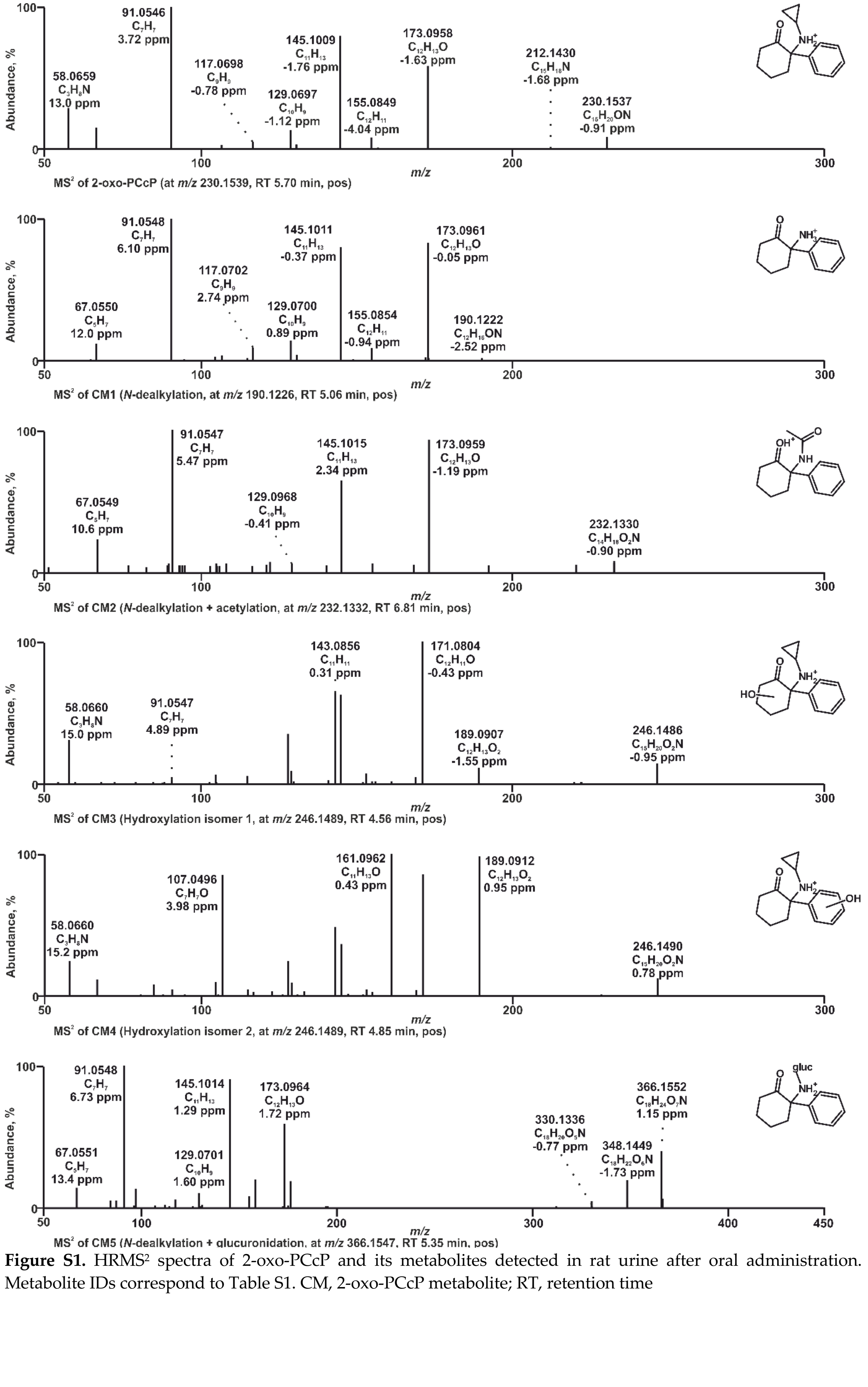

Figure S1. HRMS² spectra of 2-oxo-PCcP and its metabolites detected in rat urine after oral administration. Metabolite IDs correspond to Table S1. CM, 2-oxo-PCcP metabolite; RT, retention time

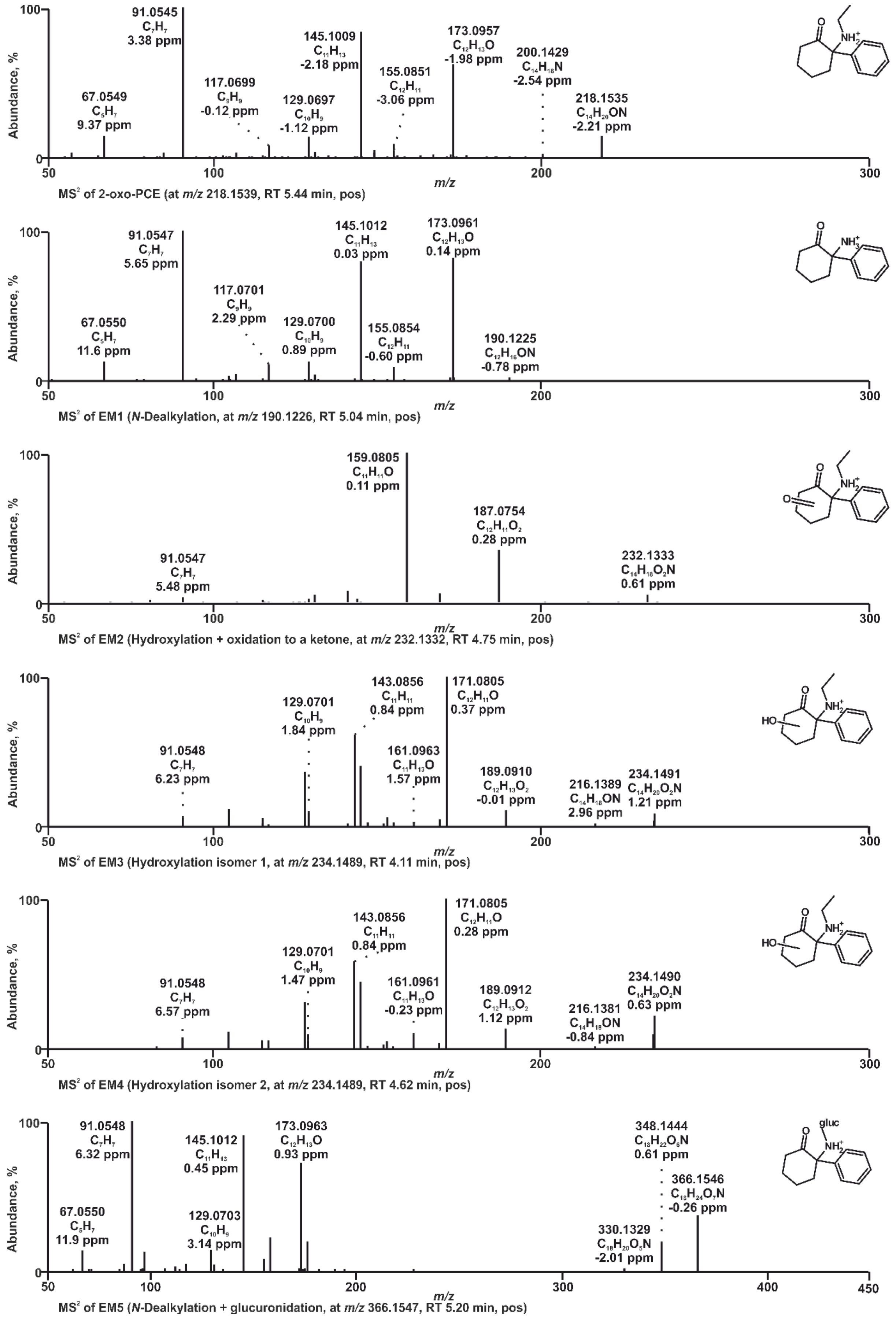

Figure S2. HRMS^2 spectra of 2-oxo-PCE and its metabolites detected in rat urine after oral administration. Metabolite IDs correspond to Table S2. EM, 2-oxo-PCE metabolite; RT, retention time

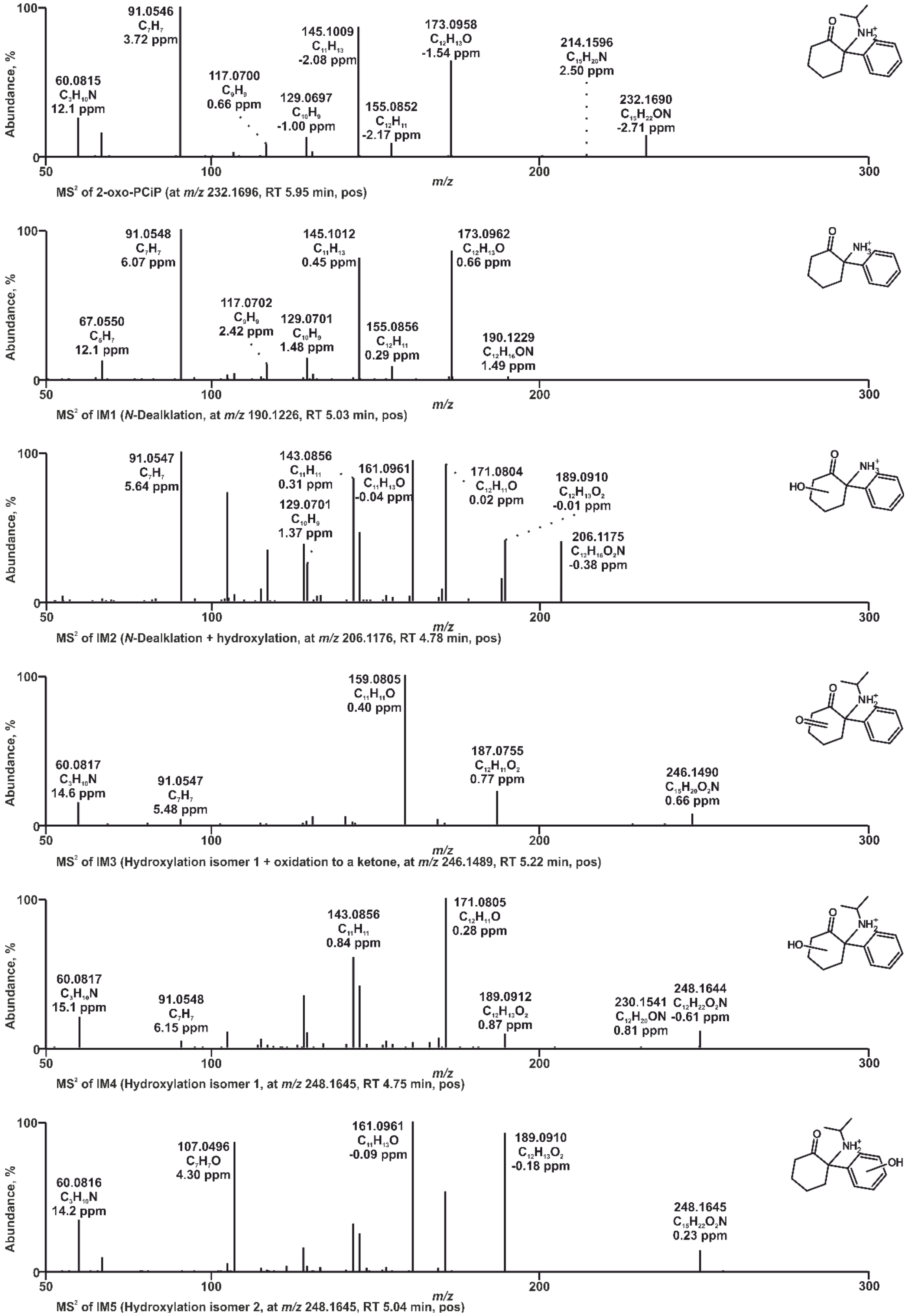

Figure S3. $HRMS^2$ spectra of 2-oxo-PCiP and its metabolites detected in rat urine after oral administration. Metabolite IDs correspond to Table S3. IM, 2-oxo-PCiP metabolite; RT, retention time

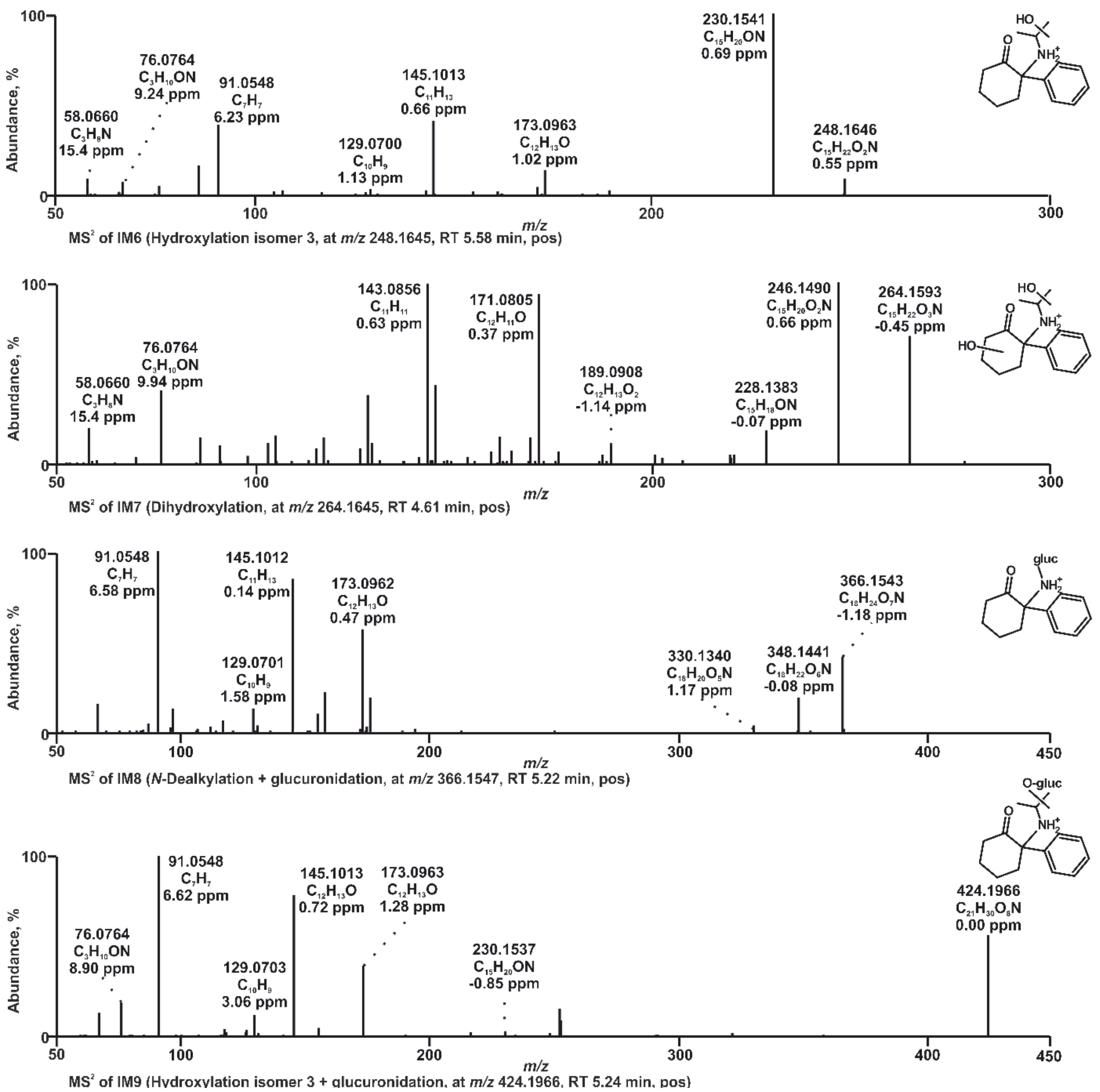

Figure S3. continued

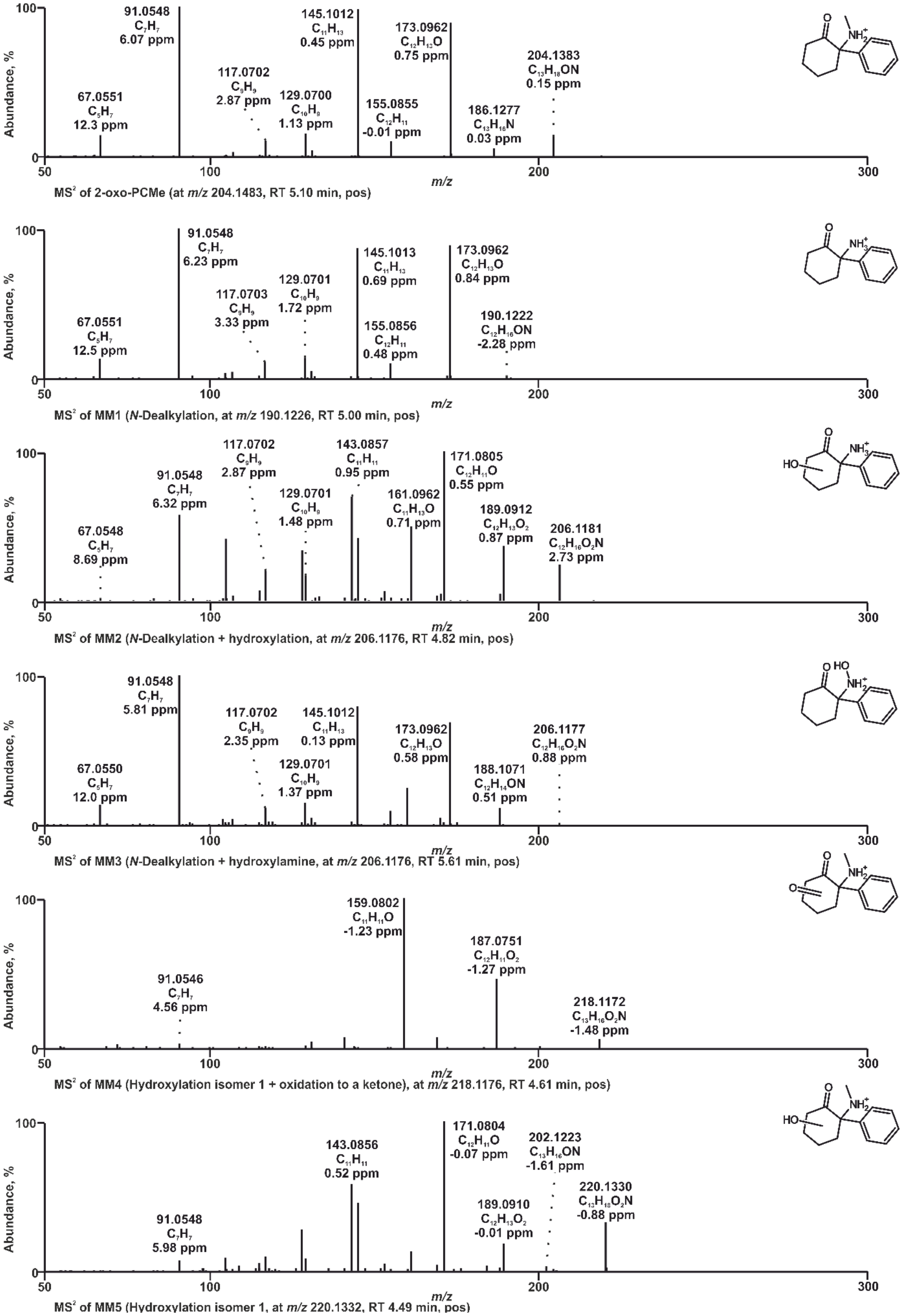

Figure S4. HRMS[2] spectra of 2-oxo-PCMe and its metabolites detected in rat urine after oral administration. Metabolite IDs correspond to Table S4. MM, 2-oxo-PCMe metabolite; RT, retention time

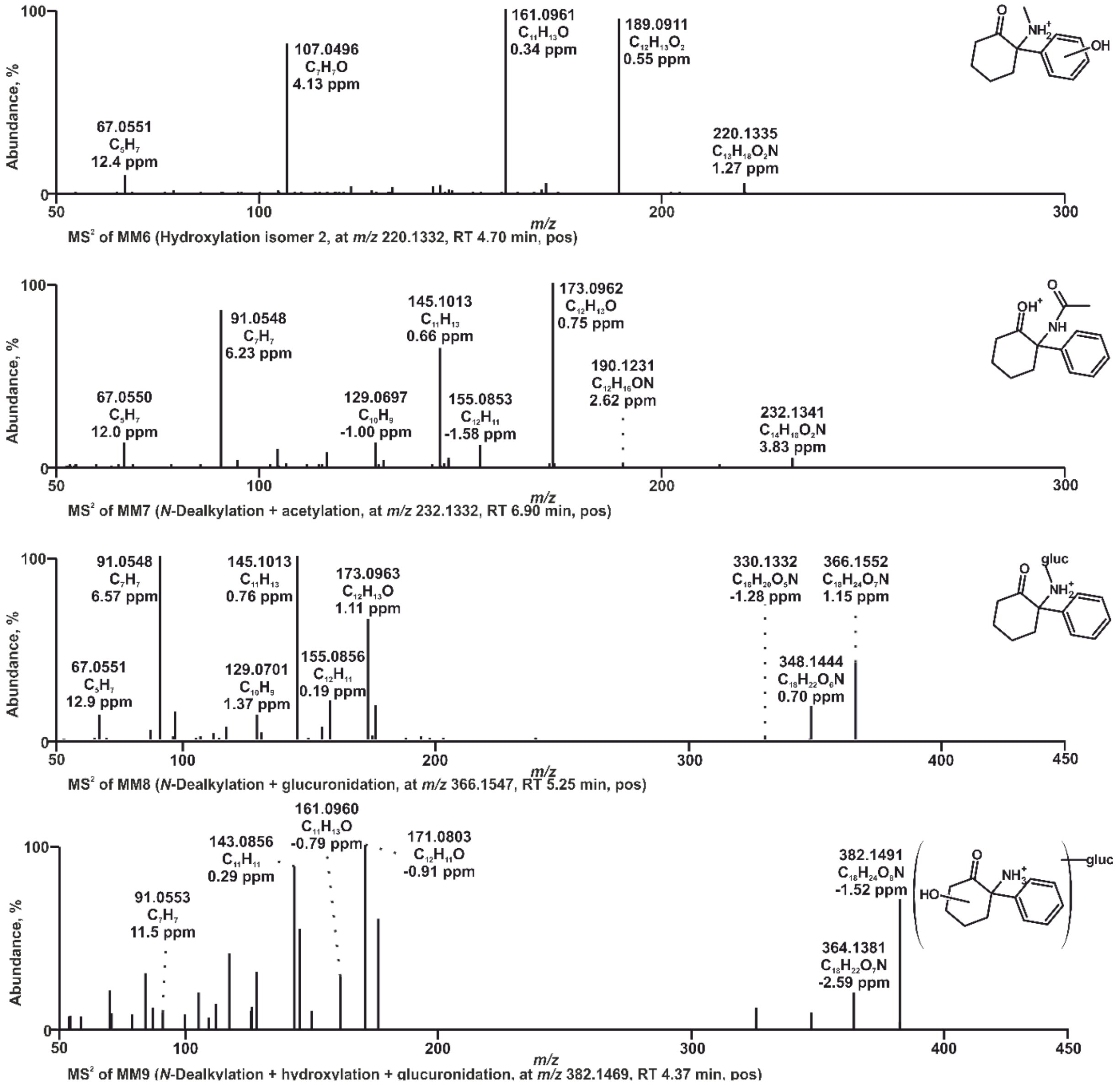

Figure S4. continued

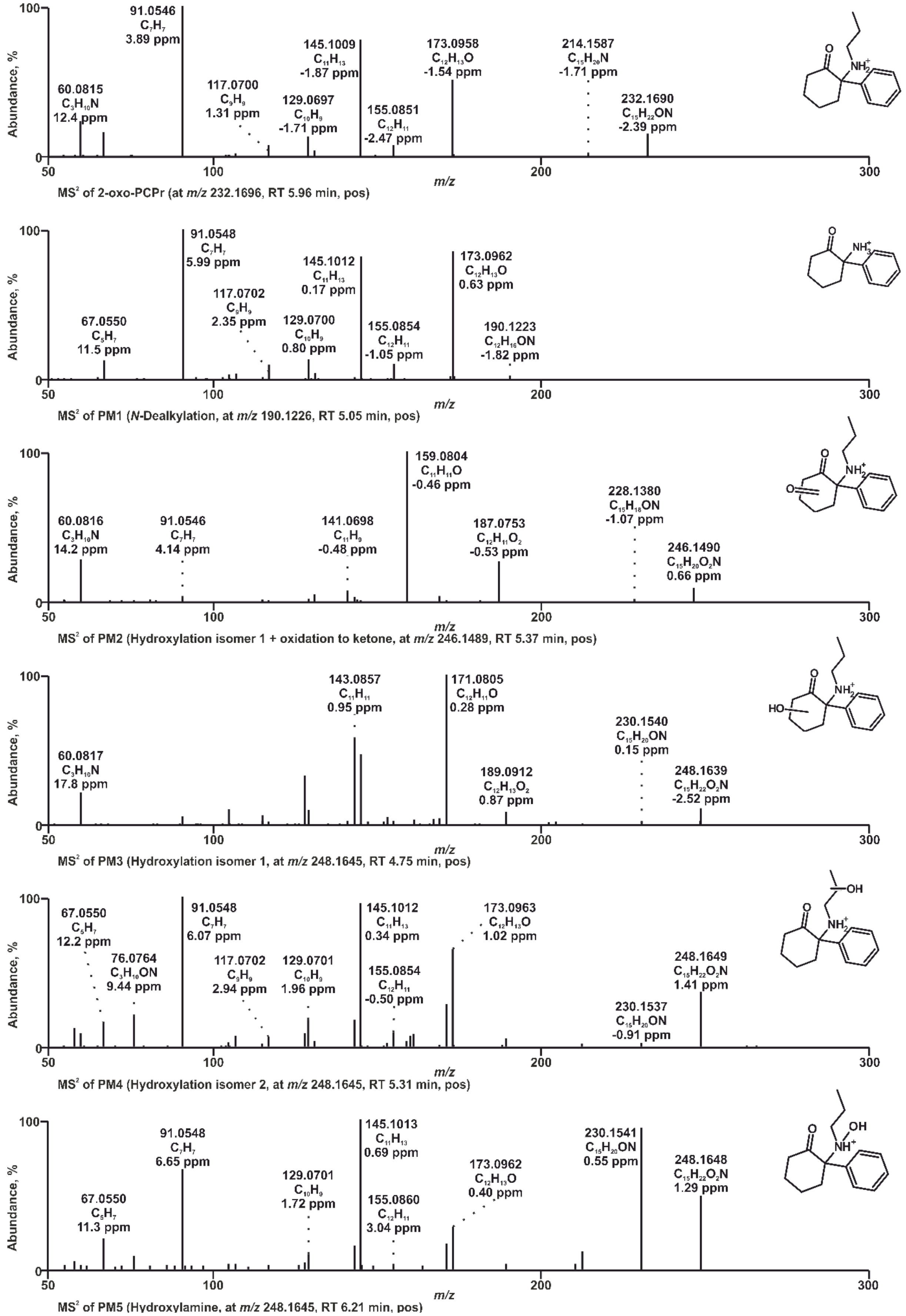

Figure S5. $HRMS^2$ spectra of 2-oxo-PCPr and its metabolites detected in rat urine after oral administration. Metabolite IDs correspond to Table S5. PM, 2-oxo-PCPr metabolite; RT, retention time

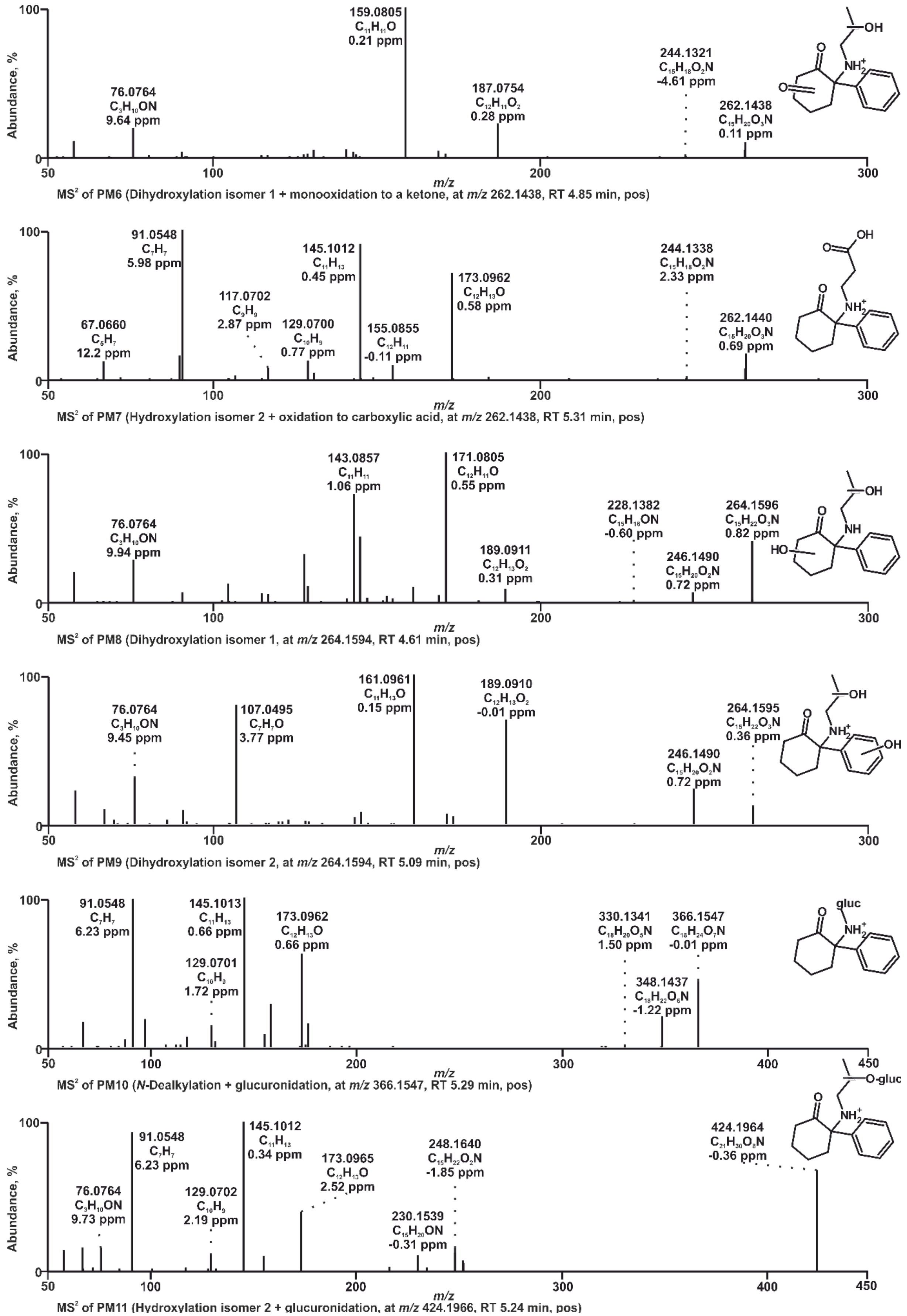

Figure S5. continued

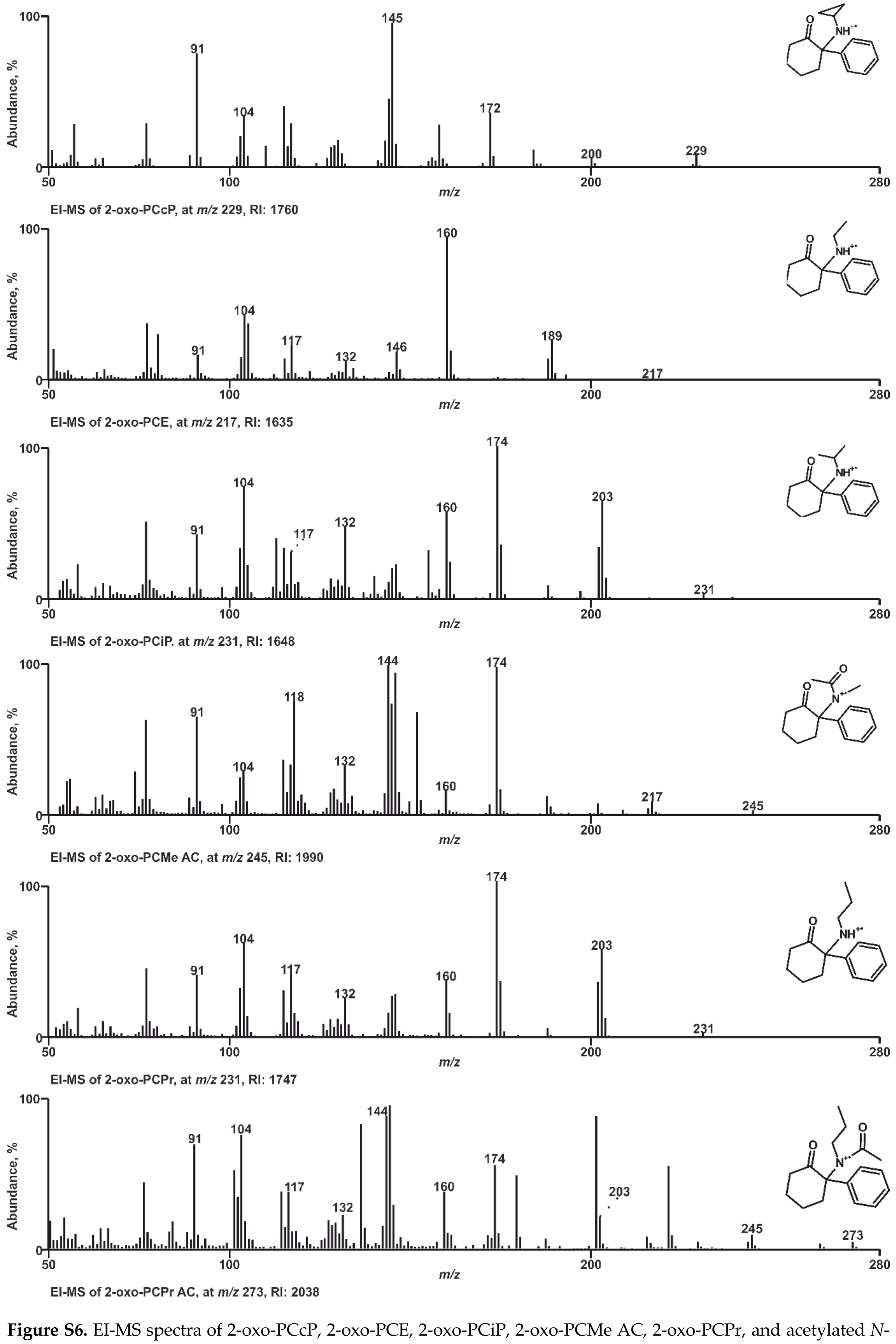

Figure S6. EI-MS spectra of 2-oxo-PCcP, 2-oxo-PCE, 2-oxo-PCiP, 2-oxo-PCMe AC, 2-oxo-PCPr, and acetylated *N*-dealkylation metabolites. Metabolite IDs correspond to Tables S1-S5. RI: retention index.

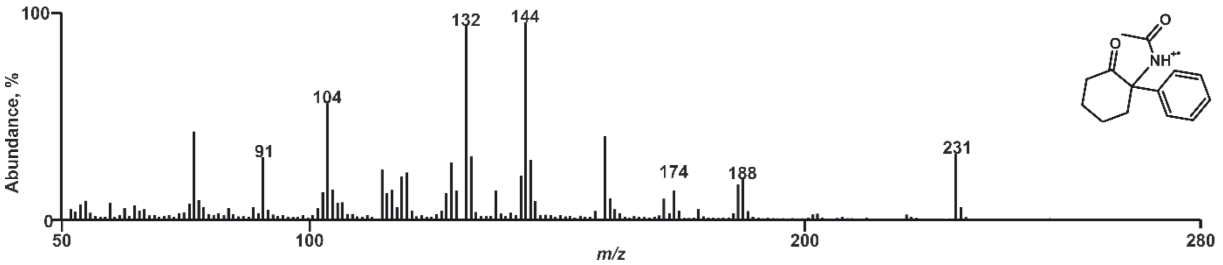

EI-MS of acetylated *N*-dealkylation metabolites CM1, EM1, IM1, MM1, and PM1, at m/z 231, RI: 1874

Figure S6. continued

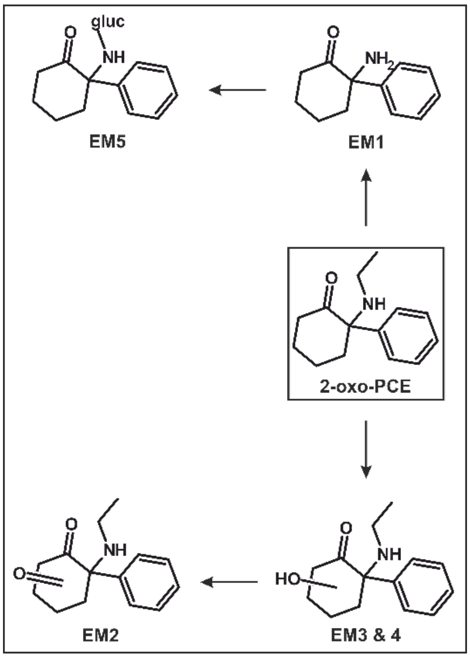

Figure S7. *In vivo* metabolic pathways of 2-oxo-PCE, ID corresponding to Table S2. EM, 2-oxo-PCE metabolite.

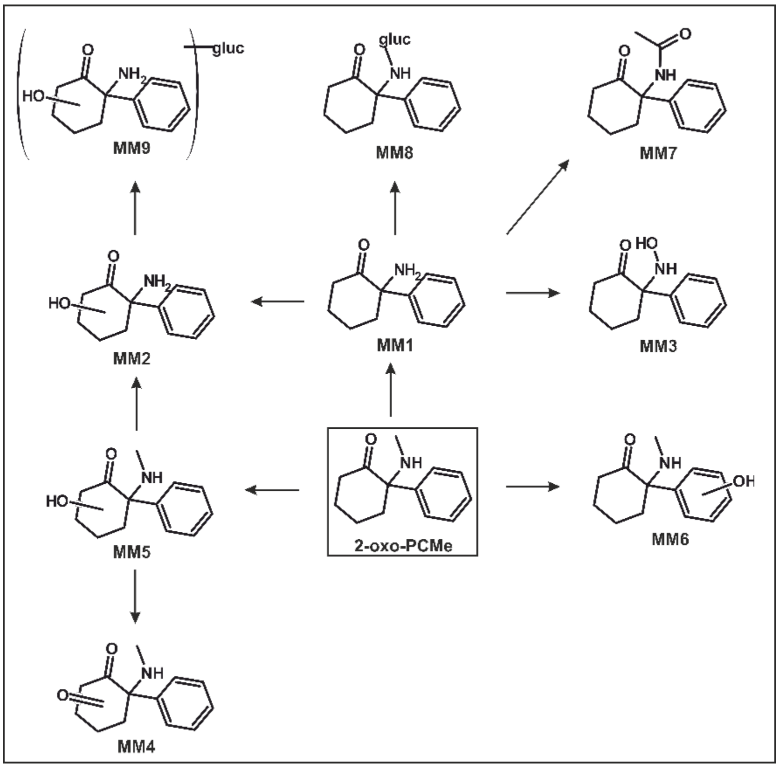

Figure S8. *In vivo* metabolic pathways of 2-oxo-PCMe, ID corresponding to Table S4. MM, 2-oxo-PCMe metabolite.
